# Evaluation of cationic channel TRPV2 as a novel biomarker and therapeutic target in Leukemia-Implications concerning the resolution of pulmonary inflammation

**DOI:** 10.1038/s41598-018-37469-8

**Published:** 2019-02-07

**Authors:** Kodappully S. Siveen, Kirti S. Prabhu, Aeijaz S. Parray, Maysaloun Merhi, Abdelilah Arredouani, Mohamed Chikri, Shahab Uddin, Said Dermime, Ramzi M. Mohammad, Martin Steinhoff, Ibrahim A. Janahi, Fouad Azizi

**Affiliations:** 1 Translational Research Institute, Academic Health System, Hamad Medical Corporation, Doha, Qatar; 2 National Center for Cancer Care and Research-Hamad Medical Corporation, Doha, Qatar; 3 Qatar Biomedical Research Institute, Qatar Foundation, Doha, Qatar; 4 Department of Oncology, Karmanos Cancer Institute, Wayne State University, Detroit, Michigan USA; 5 Division of Pediatric Pulmonology Sidra Medicine, Doha, Qatar

## Abstract

Patients treated during leukemia face the risk of complications including pulmonary dysfunction that may result from infiltration of leukemic blast cells (LBCs) into lung parenchyma and interstitium. In LBCs, we demonstrated that transient receptor potential vanilloid type 2 channel (TRPV2), reputed for its role in inflammatory processes, exhibited oncogenic activity associated with alteration of its molecular expression profile. TRPV2 was overexpressed in LBCs compared to normal human peripheral blood mononuclear cells (PBMCs). Additionally, functional full length isoform and nonfunctional short form pore-less variant of TRPV2 protein were up-regulated and down-regulated respectively in LBCs. However, the opposite was found in PBMCs. TRPV2 silencing or pharmacological targeting by Tranilast (TL) or SKF96365 (SKF) triggered caspace-mediated apoptosis and cell cycle arrest. TL and SKF inhibited chemotactic peptide fMLP-induced response linked to TRPV2 Ca^2+^ activity, and down-regulated expression of surface marker CD38 involved in leukemia and lung airway inflammation. Challenging lung airway epithelial cells (AECs) with LBCs decreased (by more than 50%) transepithelial resistance (TER) denoting barrier function alteration. Importantly, TL prevented such loss in TER. Therefore, TRPV2 merits further exploration as a pharmacodynamic biomarker for leukemia patients (with pulmonary inflammation) who might be suitable for a novel [adjuvant] therapeutic strategy based on TL.

## Introduction

Leukemia covers a broad spectrum of hematological neoplasms characterized by profound genetic alterations of the bone marrow hematopoietic precursors which transform into different types of abnormal immature blasts cells exhibiting differentiation arrest, defective apoptosis, and increased proliferative potential^1^. Ultimately, the bone marrow microenvironment is hijacked by LBCs through different not well understood molecular signaling pathways to promote cancer cells survival and spill out into the bloodstream^1,2^.

Accumulation of a large number of immature myeloid cells in [uncontrolled] leukemia can cause defects in both humoral and cellular immunity, thereby leading to impairment of the defense mechanisms of the host and contributing to the incidence of infection which is a major obstacle in the treatment of leukemia leading to life threatening situations or death^3^. Particularly, respiratory complications due to infections are considered the major cause of morbidity and mortality in the immunocompromised leukemia patients^3^.

Additionally, a bulk of data on pulmonary extramedullary manifestations in patients with leukemic disorders consists of complications due to LBCs infiltration, which can develop during the course of the disease^4–7^. Especially, patients with a high blast cell counts (up to 70 to 90%) become more vulnerable to lung inflammation and respiratory failure due often to LBCs settled in the extravascular spaces of the lungs^7^. In fact, LBCs, like hematopoietic stem cells, have similar migratory and trafficking potential^8^, and frequently acquire the capacity to spontaneously infiltrate and invade organs^4–6,9,10^. LBCs infiltration of the lung may result in alveolar damage, alteration of gas exchange, and ultimately respiratory failure and death^11^.

The lung airway epithelium forms a physical barrier against inhaled pathogens, and orchestrates immune and pulmonary inflammatory responses^12,13^. Impairment of the airway epithelium integrity and/or physiological functions may increase susceptibility to infection and other inflammatory disorders of the lung^12–15^. Hence, there is a good deal of evidence that pulmonary leukemic infiltrates may directly damage airway epithelium and induce an uncontrollable hyperinflammatory reaction in the lung. Nonetheless, the systematic investigation of LBCs interaction with AECs is currently lacking.

In this study, we brought to light a seemingly fatal complication of leukemia and a new dimension in therapy for [hard to treat] leukemia that can also be adopted for resolving [pulmonary] inflammation. To achieve this goal, we sought to identify a marker in leukemic blasts that fulfills criteria such as exhibition of oncogenic capacity, involvement in inflammatory processes (e.g. migration/extravasation), and ideally can be exploited as a therapeutic target.

The transient receptor potential vanilloid type two (TRPV2) channel emerged as a candidate channel in several deadly cancers promoting proliferation and resistance of cancer cells to apoptotic-induced cell death^16–20^. Depending on the type of cancer, loss, gain, and alternative splicing of TRPV2 gene were found to exhibit oncogenic capacity that is associated with solid tumors growth and progression. Despite a plethora of evidence showing aberrant TRPV2 expression in hematological tumors^17,21^, not much is known about its role in leukemogenesis.

TRPV2 is a mechanosensitive cation channel acting as a molecular sensor in diverse immune cells functions that include phagocytosis and degranulation^22,23^, migration (chemotaxis)^22–25^, cytokines secretion^23^, and infiltration of tissues^26^. Interestingly, TRPV2 channel is one of the molecular targets of TL, which is considered a specific blocker of TRPV2 Ca^2+^-activity^19,22,27–30^. TL (brand name *Rizaben*), is an anti-inflammatory drug approved for the treatment of allergic disorders such as bronchial asthma^31^, but also exhibits anti-fibrotic and anti-cancer activities *in vitro* and *in vivo*^31^. Additionally, in LBCs^32^, TRPV2 Ca^2+^-activity has been shown to be inhibited by SKF, which displays broad anti-cancer properties^33,34^.

In the present study, we screened different LBCs for TRPV2 expression profile, assessed TRPV2 oncogenic capacity using siRNA technology and its antagonists TL and SKF, and conducted electrophysiology experiments to examine whether LBCs affect airway epithelium structure and function under conditions that mimic the *in vivo* situation.

## Results

### TRPV2 molecular expression profile is altered in leukemic blast cell lines

**Figure 1:**
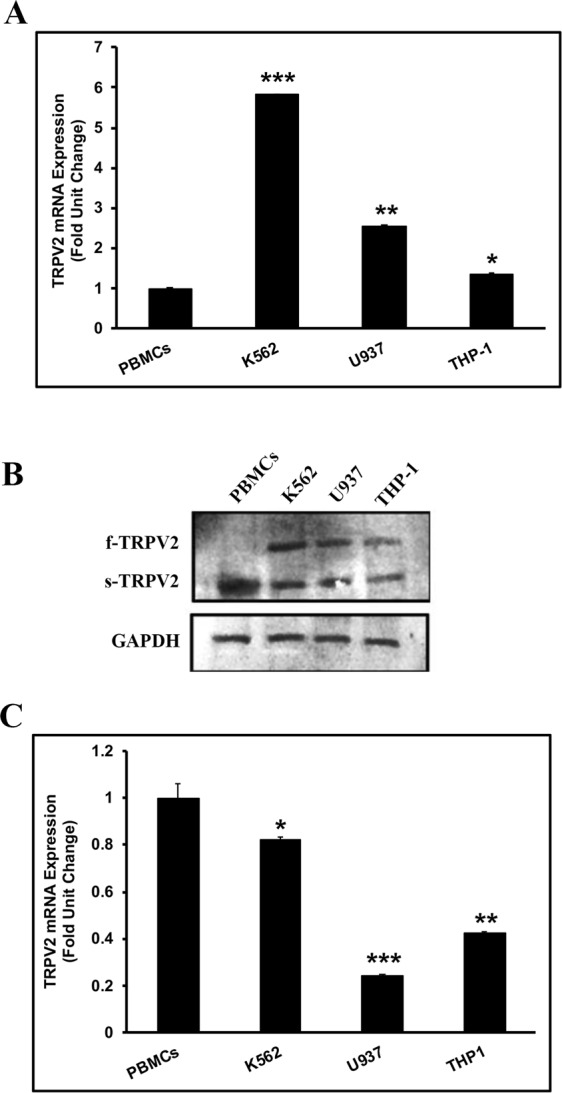
TRPV2 molecular expression profile is altered in leukemic blast cell lines. (**A**) RT-qPCR analysis of TRPV2 transcripts mRNA using a set of primers to detect all TRPV2 isoforms. TRPV2 is overexpressed in all LBCs compared to normal PBMCs ((*F*(3, 12) = 128.82, *p* < 2 × 10^−9^), *P < 0.01, **P < 0.001, ***P < 0.0005). (**B**) Western blot analysis of whole cell lysate samples using a polyclonal antibody generated against a C-terminal region common to all TRPV2 isoforms. Note the differential expression levels of s-TRPV2 and f-TRPV2 between LBCs and PBMCs. (**C**) RT-qPCR analysis of samples using a set of primers to detect specifically s-TRPV2 isoform. The highest expression level of s-TRPV2 is found in normal PBMCs ((*F*(3, 9) = 8.08, *p* < 0.006), *P < 0.02, **P < 0.002, ***P < 0.003). Data are presented as the mean ± SD, and p values indicate significant difference from control (PBMCs).

We used RT-qPCR and western blot to determine TRPV2 mRNA transcript expression level in PBMCs collected from healthy donors and LBCs K562, U937, and THP-1 defined elsewhere (see Material & Methods section). Using a set of primers designed to detect all TRPV2 isoforms, we found that total TRPV2 mRNA levels were significantly higher in LBCs compared to normal PBMCs (Fig. 1A). The highest increase (≈6-fold) in TRPV2 mRNA levels was observed in K562 cells. Strikingly, immunoblotting analysis uncovered a differential expression profile of TRPV2 protein in the different cell preparations (Fig. 1B). We identified two bands with molecular weights of ~100 kDa and ~75 kDa corresponding respectively to full length glycosylated TRPV2 protein (f-TRPV2)^35,36^, and short splice variant of TRPV2 (s-TRPV2) essentially lacking the pore forming region of the channel^24^.

Note that f-TRPV2 is significantly expressed in all the LBCs but it was barely detected in PBMCs. The strongest expression of this functional isoform was seen in K562 cells. In contrast, the non-functional s-TRPV2 isoform was much more abundant in PBMCs than in LBCs. The identity of s-TRPV2 and its down-regulation in LBCs in comparison to normal PBMCs were further confirmed using a set of primers specifically designed to uniquely detect this splice variant of TRPV2 (Fig. 1C). Therefore, alteration of TRPV2 gene expression profile may reflect its oncogenic potential in LBCs.

### TRPV2 siRNA triggered apoptotic cell death in leukemic blast cell lines

**Figure 2:**
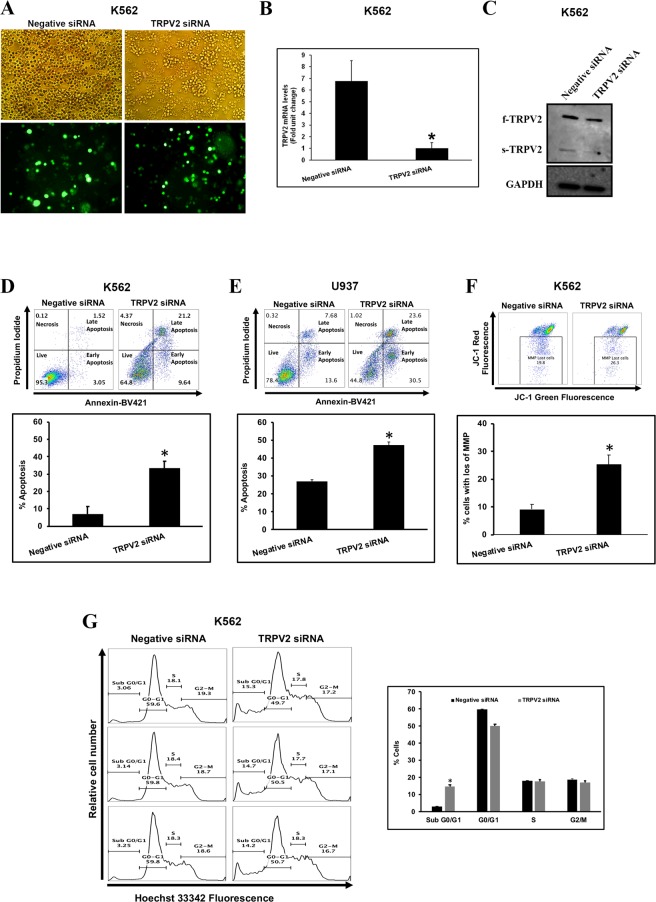
TRPV2 siRNA triggered apoptotic cell death in leukemic blast cell lines. (**A**) Transmission (Top panels) and fluorescence (bottom panels) imaging showing successful transfection of K562 cells with fluorescein-coupled small interfering RNA (siRNA) targeting TRPV2. Negative siRNA represents a non-targeting siRNA control. Efficiency of TRPV2 gene silencing was determined 24 h after transfection by (**B**) qRT-PCR (performed with the set of primers to detect all TRPV2 isoforms), and (**C**) Western blot. TRPV2 gene silencing-induced apoptosis in (**D**) K562 (*P < 0.001) and (**E**) U937 (*P < 0.005) detected by the Annexin V/7-AAD-based flow cytometry assay. (**F**) TRPV2 siRNA-induced loss of MMP in K562 detected by the JC-1-based flow cytometry assay (*P < 0.005). (**G**) TRPV2 silencing triggered cell death that is indicated by an increase in the sub G0/G1 cell population (*P < 0.001). Data are presented as the mean ± SD.

We used electroporation to transfect LBCs with fluorescein-labeled small interfering RNA (siRNA) targeted to TRPV2. After 24 h, the majority of LBCs were successfully transfected with siRNA (Fig. 2A), and cellular TRPV2 has been significantly reduced (Fig. 2B,C).

TRPV2 gene silencing triggered significant apoptosis in K562 (Fig. 2D) and U937 cells (Fig. 2E). Interestingly, K562 cells, which have the highest level of f-TRPV2, were the most affected by TRPV2 siRNA and displayed a bigger fraction of apoptotic cells. A significant loss in mitochondrial membrane potential (MMP) (Fig. 2F) indicated that TRPV2 siRNA induced cell death by activating the mitochondrial apoptotic pathway. However, we had inconclusive data regarding TRPV2 siRNA-induced apoptosis in the THP-1 leukemia cell line even after a 48 h post-transfection time period, probably because of the low expression level of f-TRPV2 in this LBC (data not shown).

TRPV2 gene silencing did not induce cell cycle arrest in K562 cells, but significantly increased the Sub G0/G1 cell population with fragmented DNA reflecting cell death and apoptosis (Fig. 2G).

Taking together, These findings indicate that the oncogenic growth and proliferation of LBCs is driven in part by alteration of TRPV2 gene expression profile.

### TL and SKF modulated TRPV2 molecular expression profile in leukemic blast cell lines

**Figure 3:**
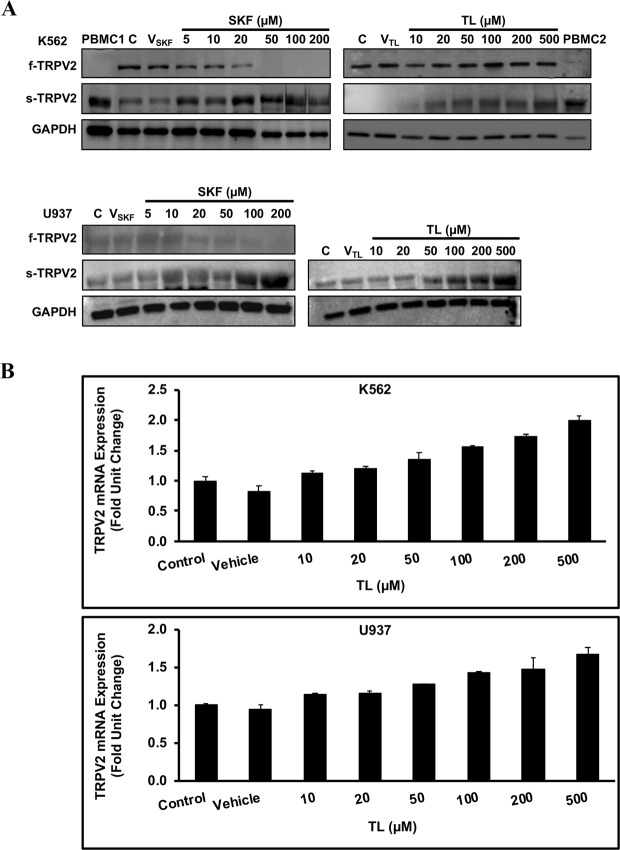
TL and SKF modulated TRPV2 molecular expression profile in leukemic blast cell lines. (**A**) Immunoblotting analysis of whole cell lysates prepared from SKF- and TL-treated LBCs unveiled a different expression profile of f-TRPV2 and s-TRPV2 protein isoforms compared to untreated cells (C, control) or cells treated either with SKF vehicle (V_SKF_, deionized water) or TLvehicle (V_TL_, DMSO). Note that s-TRPV2 expression was restored to a level comparable to that in PBMCs preparations (PBMCs 1 and 2). Refer to supplementary figures 3S1_gel 1, 3S1_gel 2, 3S2_gel 1 A, 3S2_gel 1B, 3S3_gel 1, and 3S3_gel 2 for more details. (**B**) RT-qPCR analysis of samples from TL-treated LBCs using specific primers for s-TRPV2 isoform. TL increased transcription level of s-TRPV2 in a dose-dependent manner. Data are presented as the mean ± SD.

In several patch-clamp studies, TRPV2 Ca^2+^ activity has been pharmacologically targeted by TL, an analog of a tryptophan metabolite that specifically inhibits TRPV2 Ca^2+^ activity by binding to the pore region of this channel^19,22,28–30^. SKF, an imidazole derivative drug, known as a broad spectrum inhibitor of Ca^2+^ influx through different transient receptor potential (TRP) channels^22^, was also shown to directly block TRPV2 Ca^2+^ activity^32,37,38^. The inhibitory effect of these drugs on TRPV2 Ca^2+^ activity is immediately observed upon their application. Nonetheless, we opted for longer treatments with TL and SKF to test their anti-cancer activities in LBCs. Prior to that, we treated LBCs with TL or SKF for 48 h and investigated their effect on TRPV2 expression profile. Surprisingly, TL and SKF induced a drastic change in expression levels of f-TRPV2 and s-TRPV2 protein isoforms (Fig. 3A). In K562 cells, SKF, in a dose-dependent manner, significantly evoked an increase in expression level of s-TRPV2 concomitant with a parallel decrease in expression level of f-TRPV2 (see also Supplementary Fig. 3S1_gel3). However, TL increased level of s-TRPV2 in a dose-dependent manner, but intriguingly it did not significantly alter f-TRPV2 level (see also Supplementary Fig. 3S2_gel2). Similarly, in U937 cells, TL and SKF significantly boosted the expression level of s-TRPV2 in a dose-dependent manner, thereby demonstrating the effectiveness of these drugs in restoring s-TRPV2 expression to a higher level (Fig. 3A, Supplementary Fig. 3S3_gels 1 and 2). In a dose-dependent manner, TL increased mRNA transcript levels of s-TRPV2 in LBCs (Fig. 3B). Similar results were obtained in SKF-treated LBCs (data not shown). Therefore, these data indicate that TL and SKF can reverse the aberrant expression profile of TRPV2 in LBCs, and have the ability to suppress the oncogenic activity of TRPV2 in LBCs.

### TL and SKF inhibited growth of leukemic blast cell lines

**Figure 4:**
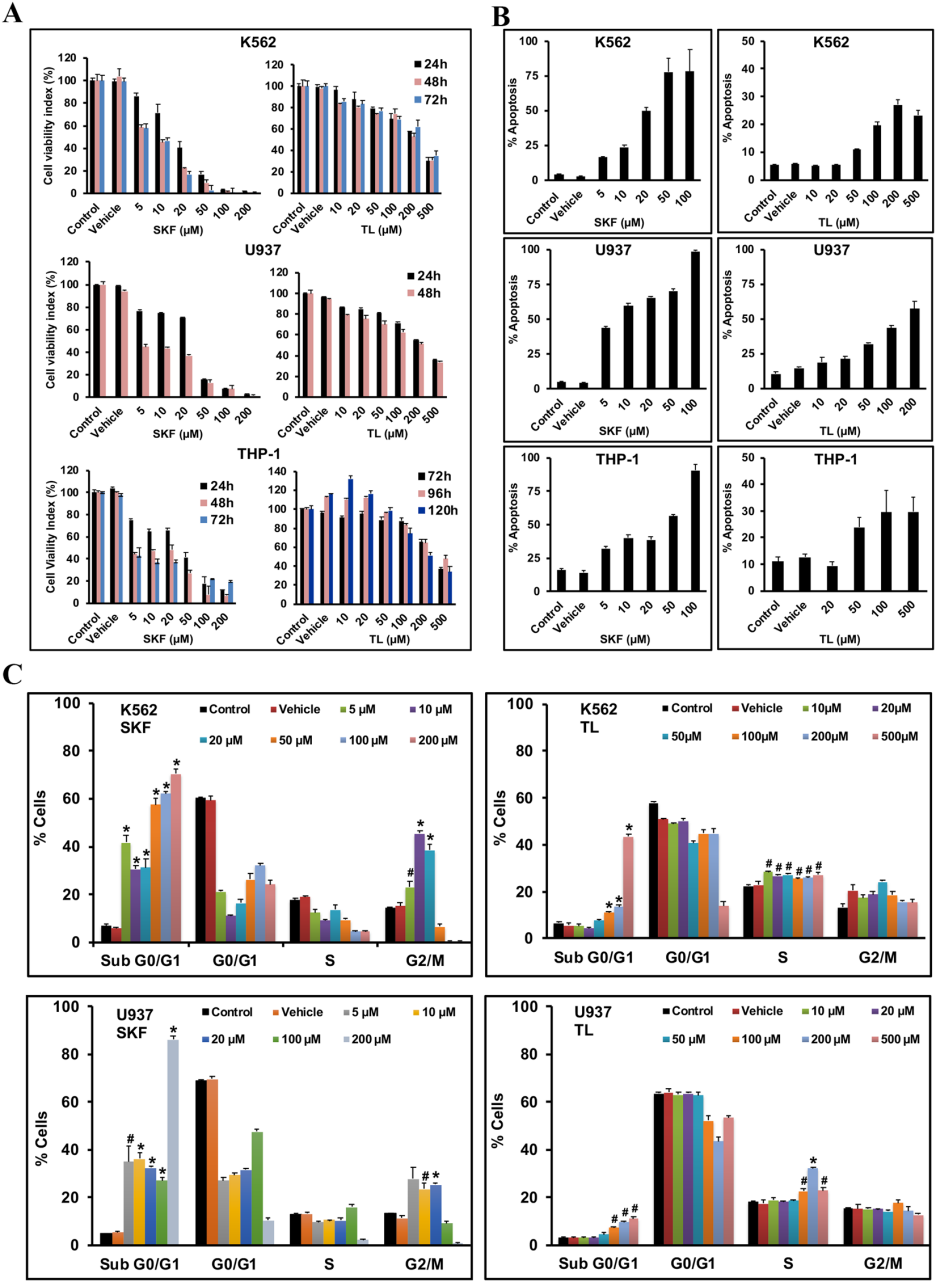
TL and SKF treatments inhibited LBCs growth via apoptosis and cell cycle arrest. (**A**) MTT proliferation assay to assess LBCs viability in response to TL (right panels) or SKF (left panels) treatments. Cell viability is expressed as a relative value to that of the untreated cells (control) which was set to 100%. (**B**) Levels of total apoptosis, representing early plus late apoptotic cell fractions, were recorded in K562 and U937 cells treated with TL or SKF for 48 h, and THP-1 treated for 120 h. (**C**) Cell cycle analysis of LBCs exposed to TL or SKF for 48 h. Proportions of cells in G0/G1, S, G2/M phases, and Sub G0/G1 phases were plotted. TL induced arrest mainly at phase S as well as a statistically significant cell death (Sub G0/G1) at all doses in both K562 and U937 cells. SKF at 5, 10 and 20 µM induced G2/M arrest, while all doses induced cell death in both K562 and U937 cells. Data displayed in the graphs represent the mean ± SD ((*F*(7, 16) = 13.7–95.9, *p* < 5 × 10^−5^). ^#^P < 0.005, *P < 0.0001, significantly different from control).

TL (IC_50_ ≈ 200 µM) and SKF (IC_50_ ≈ 10 µM) effectively inhibited the growth of all LBCs in a dose- and time-dependent manner (Fig. 4A). Nevertheless, SKF was more potent than TL, probably because SKF targets both TRPV2 isoforms and its response may arise from additional off-target effects. The anti-cancer effect of SKF was similar in all LBCs and was observed after 24–48 h of treatment. However, TL was more effective in K562 and U937 cells, both of which have higher levels of f-TRPV2 compared to THP-1. In fact, a 24 or 48 h treatment with TL was sufficient to substantially reduce K562 and U937 growth, but for THP-1 a period of 96 or 120 h was usually required to obtain a significant inhibitory effect. The IC_50_ of TL may seem to be high but it is within the range of doses that are clinically administered or used previously in the *in vitro* and *in vivo* studies^31^. Ruthenium red, a pan inhibitor of TRPV channels^22^, also caused a similar growth inhibition of LBCs but probenecid, which is known to stimulate TRPV2 Ca^2+^ influx activity^22^, did not affect LBC viability (data not shown).

These results indicate that the anti-proliferative effects of TL and SKF can be attributed at least in part to targeting TRPV2, thereby reinforcing the concept that TRPV2 may be considered as a potential pharmacodynamic biomarker.

### TL and SKF triggered cell death via apoptosis and cell cycle arrest

In all LBCs, SKF and TL significantly induced apoptosis in a concentration dependent manner, though as expected, apoptosis was more pronounced with SKF than TL (Fig. 4B). Next, we analyzed DNA content by flow cytometry to determine the effect of SKF and TL on cell cycle distribution (Fig. 4C). Similar to TRPV2 silencing, SKF and TL induced, in a statistically significant dose-dependent manner, apoptotic cell death which was reflected by a substantial dose-dependent increase in cell population in the sub G0/G1 phase. Additionally, SKF up to 20 µM caused significant cell cycle arrest mainly at the G2/M phase in LBCs, but at higher doses, SKF was cytotoxic and induced cells killing. TL arrested cell cycle predominantly at the S phase in both K562 and U937 cells.

### TL and SKF activated the mitochondrial caspase-mediated apoptotic pathway

**Figure 5:**
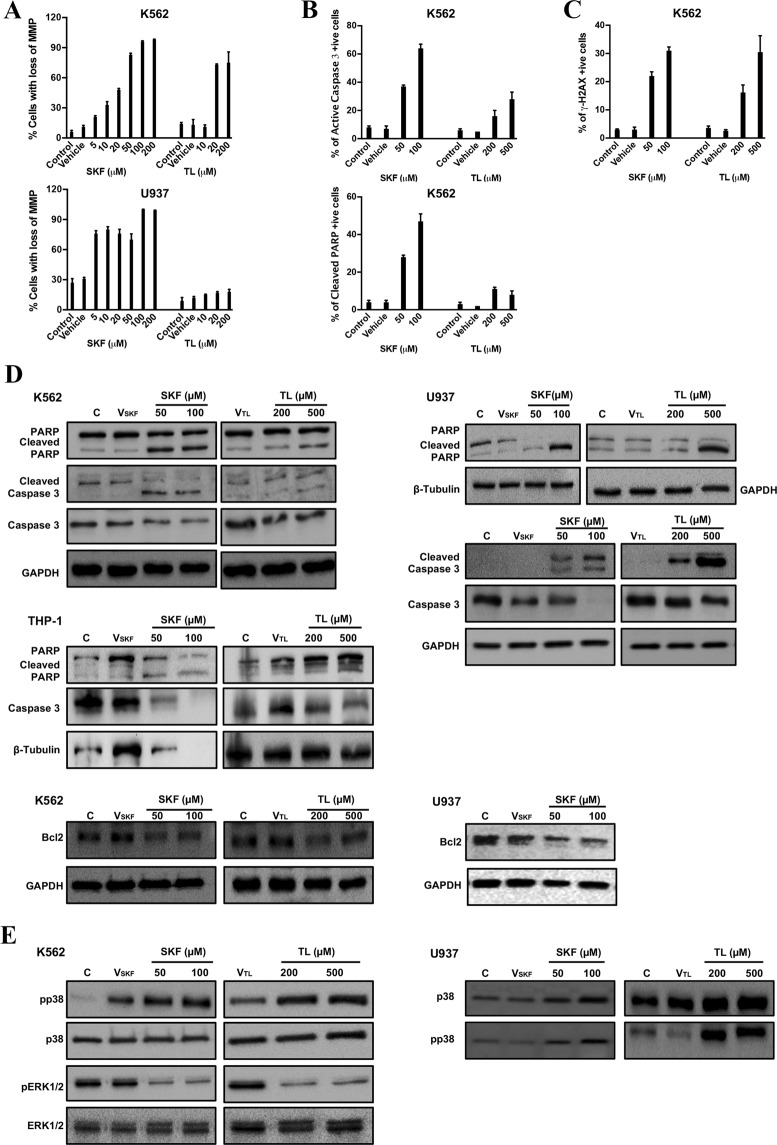
TL and SKF activated the mitochondrial caspase-mediated apoptotic pathway. (**A**) dose-dependent collapse of MMP in K562 and U937 cells treated with TL or SKF for 48 h. Cells were stained with the lipophilic cationic dye JC-1 and analyzed by flow Cytometry. (**B**) K562 were treated for 48 h with TL or SKF at the indicated concentrations, then immuno-stained and analyzed by flow cytometry to detect active (cleaved) caspase-3 and cleaved PARP levels. (**C**) Assessment of DNA damage resulting from apoptotic cell death by flow cytometry analysis of active H2AX (γ-H2AX) levels in SKF- and TL-treated K562 cells. (**D**) Whole cell lysates of TL- and SKF-treated K562, U937, or THP-1 were processed for Western blot analysis to detect PARP, cleaved PARP, caspase-3, cleaved caspase-3, and anti-apoptotic marker Bcl2. (**E**) Immunoblotting analysis of whole cell extracts to detect changes in p38, phospho (p)-38, ERK1/2, and phospho (p)-ERK1/2 in K562 and U937 cells treated with TL or SKF for 48 h. Note that p38 and ERK1/2 were activated and inactivated respectively by both drugs. Data displayed in the graphs represent the mean ± SD. Refer to Supplementary Fig. 5S for more details.

An early event of intrinsic apoptosis signaling is the loss of the MMP, which leads to the release of mitochondrial proapoptotic molecules in the cytosol and activation of the downstream caspase cascade^39^. Especially, caspase-3 considered to be the most important of the executioner caspases, and one of its substrates Poly (adenosine diphosphate-ribose) polymerase-1 (PARP), an enzyme involved in DNA repair, are considered a hallmark of apoptosis^39^. SKF and TL treatments, in a dose-dependent manner, induced activation of the mitochondrial apoptotic death pathway by causing concomitant collapse of MMP (Fig. 5A), increased levels of active caspase-3 and inactive PARP, and decrease of the anti-apoptotic factor Bcl-2, which regulates the activation of caspase proteases (e.g. caspase-3)^39^ (Fig. 5B,D).

Fragmentation of genomic DNA occurs as a late event upon induction of the caspase pathway, and triggers activation of H2AX, a key factor in the repair process of damaged DNA^40^. The double stranded breaks (DSBs) which are lethal to cells induce phosphorylation of H2AX on the 139^th^ serine residue (termed γ-H2AX)^40^. Hence, we used the flow cytometry γ-H2AX-based assay to assess DNA damage resulting from apoptotic cell death. Indeed, SKF and TL treatments resulted in a significant increase in γ-H2AX +ive cells when compared to control cells (Fig. 5C).

### TL and SKF targeted the p38-MAPK and ERK-MAPK signaling pathways

The mitogen-activated protein kinases (MAPKs) p38 and ERK1/2 are important signal transduction pathways involved in drug-induced apoptosis^41^. In general, p38 activation is associated with apoptosis induction, whereas ERK activation favors proliferation and promotes cell survival^41^. In untreated cells, some baseline phosphorylation of p38 was detectable, and such phosphorylation was substantially increased in SKF- and TL-treated LBCs (Fig. 5E). Moreover, SKF and TL treatments induced dephosphorylation of ERK1/2 kinases in a dose dependent manner when compared to control. These data demonstrate the involvement of p38-MAPK and ERK1/2-MAPK in LBCs apoptotic death elicited by TRPV2 antagonists.

### Effect of TL and SKF on intracellular Ca^2+^ levels in leukemic cell lines

**Figure 6:**
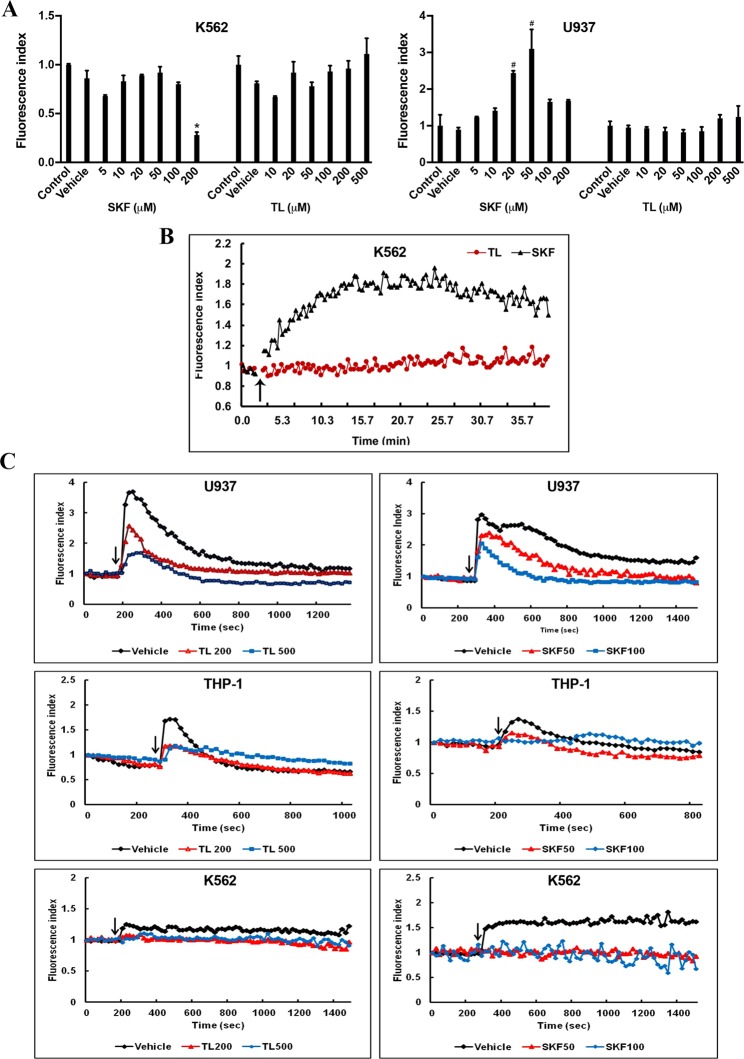
Effect of TL and SKF on Ca^2+^ Homeostasis and fMLP-evoked Ca^2+^ response in LBCs. (**A**) K562 or U937 cells were treated with TL or SKF at the indicated doses for 48 h, loaded with Fluo-4 AM, and then analyzed by flow cytometry. Data displayed in the graphs represent the mean ± SD (for K562, F(7, 14) = 84.4, P < 2 × 10^−10^; for U937, F(7, 14) = 36.3, p < 6 × 10^−8^, *P < 0.001, ^#^P < 0.01, significantly different from control). (**B**) K562 cells were loaded with Fluo-4 AM, and changes in [Ca^2+^]_i_ levels triggered before and upon application of TL (200 µM) or SKF (50 µM) (as indicated by the arrow) were monitored by flow cytometry. (**C**) K562 or U937 were exposed to TL or SKF for 48 h at the indicated concentrations, loaded with Fluo-4 AM, and changes in levels of [Ca^2+^]_i_ elicited by fMLP were monitored by flow cytometry.

It has been well established that perturbation of intracellular Ca^2+^ ([Ca^2+^]_i_) compartmentalization, can cause cytotoxicity and trigger apoptotic cell death^42^. Hence, we investigated whether a 48 h treatment with SKF and TL affected [Ca^2+^]_i_ homeostasis. LBCs were loaded with Fluo-4 AM and the Ca^2+^ signal was assessed by flow cytometry (Fig. 6A). In K562 cells, apparently none of the drugs induced a significant change in [Ca^2+^]_i_ level except that SKF at 200 µM induced a statistically significant decrease (≈70%) in Ca^2+^ level due likely to a cytotoxic effect. However, in U937 cells, SKF, up to 50 µM, evoked a significant increase in [Ca^2+^]_i_ in a dose-pendent manner, whereas TL did not significantly affect [Ca^2+^]_i_.

Interestingly, when we monitored Ca^2+^ signal immediately after exposing K562 cells to SKF at 50 µM, we observed a biphasic response of Ca^2+^ signal reflected by an exponential increase followed by a slow decrease in [Ca^2+^]_i_ (Fig. 6B). In contrast, the change in [Ca^2+^]_i_ induced by TL at 200 µM could not be detected probably due to low basal TRPV2 Ca^2+^ activity and poor sensitivity of the calcium sensitive dye.

These data indicate that SKF modulated [Ca^2+^]_i_ more than TL, thereby providing another clue for having more pronounced apoptosis with SKF than with TL.

### TL and SKF inhibited fMLP-induced Ca^2+^ response in leukemic blast cells

Infiltration of tissues by immune cells are initiated by chemotaxis and migration steps that are governed by molecular events involving chemoattractants^42^. The fMLP peptide is a well-known bacterial chemotactic factor that was found to elicit immune cells migration through stimulation of TRPV2 Ca^2+^ activity^23,24^. Here, we used flow cytometry to monitor time course changes of [Ca^2+^]_i_ in LBCs stimulated by fMLP and the readouts are shown in Fig. 6C. In untreated LBCs pre-loaded with Fluo-4AM, application of 10 µM fMLP triggered a biphasic Ca^2+^ response. During the first phase, stimulation caused a rapid increase in [Ca^2+^]_i_, which was immediately followed by a 2^nd^ phase during which a sustained increase in [Ca^2+^]_i_ was observed. fMLP elicited a very good response in U937 cells, whereas the peptide was less potent by approximately two orders of magnitude in the other two cell leukemia cell lines. This is likely due to difference in the number of fMLP receptors expressed in these LBCs. As expected, TL and SKF treatments significantly blunted the kinetics of the fMLP-evoked fast and sustained rise in [Ca^2+^]_i_ in all leukemia cell lines due to inhibition of fMLP-induced stimulation of TRPV2 Ca^2+^ activity. Subsequently, fMLP-induced LBCs migration and chemotaxis could be altered by TL and SKF.

### TL and SKF reduced expression of cell surface marker CD38 in Leukemia blast cell lines

CD38 is a cell-surface marker that promotes survival and proliferation of leukemia cells^43^, and has recently gained attention as a hallmark of inflammation in both leukemia^43^ and lung airway disease^44^. In particular, CD38 supports immune cells migration and chemotaxis^44,45^.

We used flow cytometry to monitor CD38 phenotypic changes only in the live population of LBCs treated with SKF or TL. As previously reported, THP-1 cells had a higher basal CD38 expression level (≈99%) than U937 cells (≈48%)^46^, and K562 cells expressed a very low level of this surface antigen^46^ (Table 1). A 48 h treatment with TL or SKF significantly reduced CD38 expression in U937 cells. In contrast, SKF slightly reduced CD38 level in THP1 cells and TL had no effect on this surface marker. We cannot provide a rational explanation for this unexpected differential response but perhaps it is due to a difference in the genetic makeup of the two leukemia cell lines and the fact that THP-1 cells displayed a low level of f-TRPV2, the primary target of TL and SKF. Despite the low expression of CD38 in K562, there is a tendency towards reducing levels of this marker by SKF and TL. Notably, TL-induced decrease in CD38 was associated with more apoptosis in U937 than in the other leukemia cell lines. Therefore, inhibition of LBCs survival and proliferation by TL may involve down-regulation of CD38. Based on these results, we may predict that reduced expression of CD38 in LBCs could affect their mobility and infiltration into organs, thereby reducing inflammation and morbidity.

**Table 1:** Effect of TL and SKF on CD38 expression in leukemic blast cell lines.

	% CD38 (*K562*)	% CD38(*U937*)	% CD38(*THP-1*)
Control	8.22	48.2	99.6
V_SKF_	11.4	59.2	99.5
SKF_50_	**3.3**	**25.2**	90.1
SKF_100_	**0.5**	**13.2**	**80.4**
V_TL_	6.5	52.3	99.9
TL_200_	**2.8**	**38.7**	99.8
TL_500_	**3.1**	**10.5**	99.9

Leukemia cell lines were treated with TL or SKF for 48 h (K562 and U937) or 96 h (THP-1), immunostained with fluorescently labeled anti-CD38 antibody, and analyzed by flow cytometry to detect surface marker CD38. Only live cell populations were analyzed in each condition. Data (expressed as percentage of cells expressing CD38) were computed from a representative experiment.

### Analysis of bioelectrical properties of airway epithelial cells co-cultured with leukemic blast cell lines

**Figure 7:**
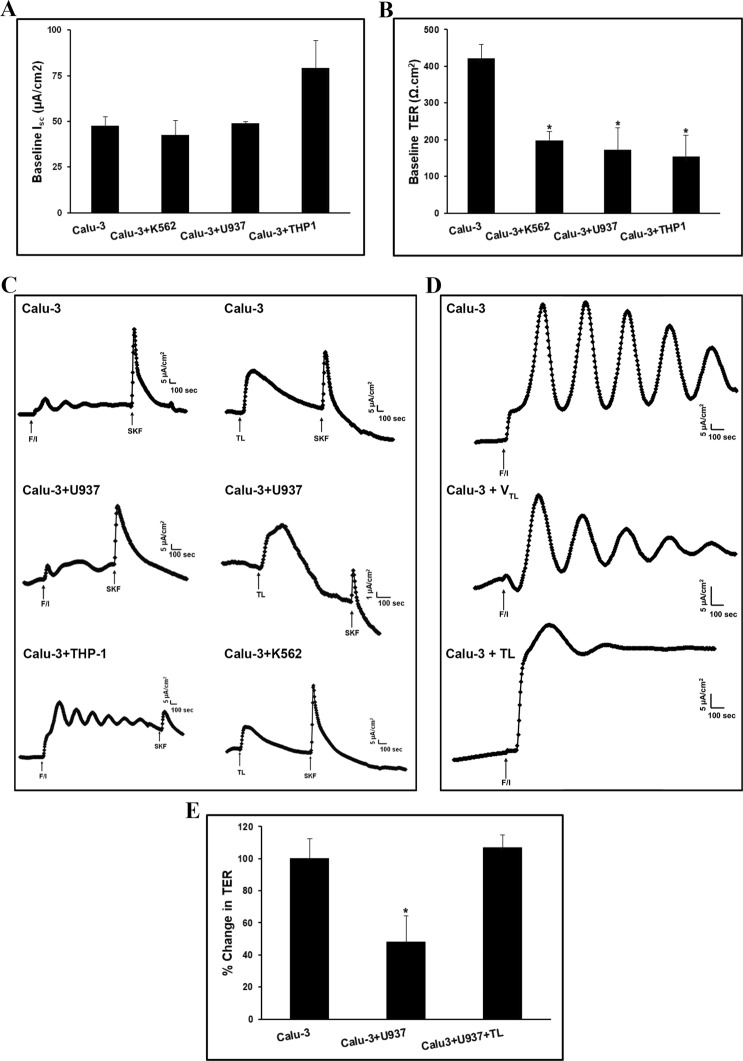
Analysis of bioelectrical properties of airway epithelial cells co-cultured with leukemic blast cell lines. Differentiated Calu-3 cells grown under air-interface conditions were further cultured alone (N = 16), or co-cultured with K562 (N = 7), U937 (N = 6), or THP-1 (N = 6) for 48 h and their I_sc_ (**A**) and TER (**B**) were assessed in Ussing chambers. Data displayed in the graphs represent the mean ± SEM (no statistical significant differences between groups’s I_sc_ (*F*(3, 25) = 2.23, *p* = 0.11); for TER (*F*(3, 27) = 7.02, *p* < 0.001), *P < 0.007, **P < 0.003, ***P < 0.001, significantly different from control (calu-3)). (**C**) Representative records of I_sc_ displaying F/I-, SKF-, and TL-evoked stimulation of active ion transport across AECs cultured alone or co-cultured with LBCs K562, U937, or THP-1 for 48 h. Drugs were applied sequentially as indicated in the I_sc_ traces either to apical (F/I, SKF) or basolateral (TL) sides of the Calu-3 monolayers. F/I, SKF, and TL were used at 10 µM/100 µM, 25 µM, and 200 µM respectively. (**D**) Representative traces of I_sc_ demonstrating F/I-evoked stimulation of active ion transport across monolayer cultures of differentiated calu-3 in absence (control) and presence of either TL vehicle (V_TL_) or TL at 500 µM for 48 h. (**E**) Differentiated calu-3 cells were co-cultured with U937 cells in absence or presence of TL for 48 h prior to assessing their TER in Ussing chambers. Data displayed in the graphs represent the mean ± SEM.

The airway epithelium is the first line of defense forming a highly-regulated barrier that protects the airways and more distal lung from injury and infection^12–15^. Here we mimicked the *in vivo* lung parenchymal/interstitial infiltration of LBCs by using the transwell culture system which has two compartments physically separated by a 0.4 μm microporous membrane to minimize direct contact between the two cell types. Surprisingly, co-culture of AECs with LBCs did not significantly alter basal I_sc_ which means there was no change in the active ion transport across AECs under resting conditions (Fig. 7A). However, challenging AECs with LBCs resulted in a marked decline (by more than 50%) in baseline TER (an index of tight junctions formation) indicating a loss of electrical barrier integrity (Fig. 7B).

Although I_sc_ response to c-AMP agonists forskolin/IBMX (F/I) and activators of the cystic fibrosis transmembrane conductance regulator (CFTR) was preserved in AECs exposed to LBCs (Fig. 7C), the magnitude of this response was significantly decreased in comparison to control (Supplementary Table 1S). Interestingly, TL and SKF, when applied respectively to mucosal and serosal sides of the cell monolayer, transiently stimulated active ion transport across control AECs or AECs co-cultured with LBCs (Fig. 7C, Supplementary Table 1S). Furthermore, we noticed that F/I, SKF, and TL were able to trigger a response regardless of their order of application, suggesting stimulation of ion transport through different signaling pathways.

Next, we examined the ability of TL to prevent the detrimental effect of LBCs on AECs barrier function. An initial assessment of AECs bioelectrical properties by Ussing chambers after a 48 h treatment with TL showed that AECs are viable and exhibited a basal I_sc_ that responds to F/I similarly to untreated control cells (Fig. 7D) except that the oscillations (waves) of I_sc_, which usually reflect changes in [Ca^2+^]_i_ level in these cells, appeared to be affected by this drug. Importantly, there was no decline in TER when AECs were co-cultured with LBCs in presence of TL (Fig. 7E), indicating that TL efficiently prevented LBCs-induced loss in AECs barrier integrity.

## Discussion

In this study, we took the first steps towards exploring whether TRPV2 can be used as a pharmacodynamic biomarker for leukemia and associated pulmonary inflammation. The choice of this channel was based on its suspected oncogenic activity in leukemia and its pleiotropic functions in immune inflammatory processes. Herein, we provide evidence that TRPV2 mRNA transcripts and protein expression profiles are altered in LBCs compared to normal PBMCs in which TRPV2 is the most abundantly expressed among TRPV family members^47^.

Silencing of TRPV2 gene demonstrated that this channel does exhibit an oncogenic activity in LBCs. Similarly, TRPV2 mRNA expression was found to be ≈12 times higher in aggressive late stage metastatic human prostate cancer samples than in localized specimens, and TRPV2 silencing reduced prostate tumor development in a xenograft mouse model^20^. In another comparative study, overexpression of TRPV2 in esophageal squamous cell carcinoma tissues, especially in the advanced stages of the disease, was found to correlate with poor prognosis of patients with this disease^48^. In contrast, silencing TRPV2 in the glioma cell line U87MG was found to increase resistance to apoptotic cell death and promotes cell proliferation and survival^49^. Hence, TRPV2 expression seems to positively or negatively regulate cancer cell growth and proliferation depending on cellular context.

Our studies provide evidence that transcriptional regulation (i.e. alternative mRNA processing) of TRPV2 is altered in LBCs. Indeed, f-TRPV2 and its short splice variant s-TRPV2 show opposite trends of expression between LBCs and normal PBMCs. Interestingly, a similar opposite trend of expression of these two isoforms was observed between normal urothelium and patients’ urothelial carcinoma tissues during advanced and aggressive stages of bladder cancer^18,22^. Hence, the TRPV2 related pathophysiological situation in LBCs examined here is comparable to the clinical pathophysiological state caused by the differential expression of the two TRPV2 isoforms in bladder cancer.

Although the presence of the short splice variant (s-TRPV2) protein was not confirmed in this previous work, removal of exons 10-11 would produce a protein without residues 529–663 corresponding to the S5-S6 segments which include the pore region of the channel^18,22^.

The s-TRPV2 identified in our study has a molecular weight of 75 kDa and is likely to correspond to the s-TRPV2 that was identified in macrophages^18,24^. This splice variant lacks the transmembrane segment 5 (S5), the pore helix and S6 which together form a functional pore in the assembled tetramer structure of the TRPV2 and would result in a protein of 74 kDa. While the [patho]physiological relevance of this splicing process is still not well understood, overexpression of the short pore-less isoform s-TRPV2 was found to impair f-TRPV2 translocation to the plasma membrane and inhibits its Ca^2+^ activity^24^. Similarly, it was reported that neurons of the rat dorsal root ganglia express a short splice variant of TRPV1 which has the ability to oligomerize with full length TRPV1 and abolish its Ca^2+^ activity^50^. The same regulatory mechanism is adopted by the short-form of TRPM2 (TRP melastatin type 2, TRPM2-S), a pore-less nonfunctional isoform of TRPM2 channel, that was shown to interact with full-length TRPM2 isoform (TRPM2-L) and negatively regulate its Ca^2+^ influx activity^51^.

Remarkably, for the first time, our data show that TL and SKF, usually used as antagonists of TRPV2 channel Ca^2+^ activity, modulate TRPV2 gene expression profile in LBCs. Interestingly, while TL affected mainly the expression profile of s-TRPV2 variant, SKF upregulated and downregulated the expression of s-TRPV2 and f-TRPV2 respectively. Herein, in a dose-dependent manner, the expression level of s-TRPV2 was fully restored by both drugs, and f-TRPV2 level was strongly reduced by SKF. Therefore, the anti-cancer effects of SKF- and TL on LBCs may be attributed in part to their ability to regulate TRPV2 gene expression by a mechanism that has yet to be elucidated. The abnormal TRPV2 expression profile in LBCs may offer a survival advantage, such as resistance to apoptotic-induced cell death that can be reversed by TL and SKF. Thus, strong evidence points toward a role for TRPV2 in the survival and proliferation of LBCs, and these data further support the concept of using TRPV2 as a pharmacodynamic biomarker for leukemia.

Our results demonstrate that targeting TRPV2 with siRNA or with its antagonists TL and SKF induced cell death by apoptosis and cell cycle arrest. The siRNA and TL experiments indicate that the degree of apoptosis somewhat correlates with the level of f-TRPV2 isoform since the leukemia cell line K562, which has the highest level of f-TRPV2, exhibited slightly more apoptosis than the U937 cell line. The THP-1 leukemia cell line, predominantly expressing the inactive s-TRPV2 isoform, seems to be insensitive to TRPV2 silencing, and three to four days were required to detect significant TL-induced apoptosis. In contrast, SKF-induced apoptosis levels were similar in the three leukemia cell lines and they were high compared to siRNA and TL. SKF exhibited more anti-cancer activity than TL likely because of its ability to negatively regulate f-TRPV2 expression, and alter [Ca^2+^]_i_ homeostasis. Its dual effect on [Ca^2+^]_i_ observed here has been previously demonstrated in the human promyeloid leukemia cell line HL60^33^. Moreover, SKF is known to target other TRP channels^22^ and store operated Ca^2+^ entry (SOCE)^34^, which may potentiate its apoptotic effect. Nevertheless, assessment of the anti-cancer effect of SKF contributed by other TRP channels (SOCE) may be difficult, if not impossible because TRP channels have different sensitivities to SKF and may occasionally form heteromers composed of different TRP channel subunits that can affect their functional effects.

Therefore, our observation that TRPV2 modulation by SKF treatment suppresses LBCs growth argues in favor of the possibility that TRPV2 is involved in the mechanism of cell death by this drug but it is not the sole mechanism and does not rule out other redundant pathways.

In line with previous studies, induction of apoptosis by TL^31^ and SKF^33^ was dependent on mitochondrial dysfunction which was characterized by MMP dissipation, activation of the mitochondrial executioner pathway, generation of cleaved caspase-3 and cleaved PARP-1 species, decrease of the anti-apoptotic factor Bcl-2 expression, DNA damage, and breakdown of phosphatidylserine asymmetry of the cell membrane. p38, known to negatively regulate Bcl-2 and ERKs^41^, was activated whereas Erk1/2 was deactivated by TL and SKF. Hence, these pathways contributed to the generation of the anti-leukemic effects of these drugs, thereby indicating a potential link between TRPV2 and these signaling pathways in the control of leukemic cells survival and proliferation. How and to which extent a crosstalk occurs between the different isoforms of TRPV2 and these signaling pathways are intriguing questions that may merit further investigation.

The alteration of TRPV2 gene expression by TL and SKF greatly reduced LBCs response to the bacterial derived chemotactic peptide fMLP, which stimulates Ca^2+^ influx through TRPV2 channels to induce cell chemotaxis and migration^24^. Subsequently, inhibition of TRPV2-dependent mobility and chemotaxis processes^23,24,52^ will greatly impact organ infiltration by LBCs and reduce tissues inflammation^26^.

TRPV2 is a homotetrameric N-glycosylated protein with plasma membrane and intracellular pools that are regulated mainly by the phosphatidylinositol 3-kinase (PI3K)^22,30^. Previousely, Nagasawa and co-workers^24^ demonstrated that fMLP-induced migration of mouse TtT/M87 macrophages specifically required PI3K-dependent trafficking of TRPV2 from the internal pool to cell surface and Ca^2+^ influx through this channel. Interestingly, overexpression of the pore-less s-TRPV2 in this cell line abrogated TRPV2 Ca^2+^ activity by inhibiting its translocation to plasma membrane, thereby affecting the process of migration which was also inhibited by ruthenium red, a pan inhibitor of TRPV channels^24^. Likewise, SKF and TL, through up-regulation of s-TRPV2, may abolish LBCs chemotactic responsiveness to prevent or attenuate [pulmonary] tissues infiltration and inflammation, especially in undesired situations where LBCs are either activated by inflammatory mediators supplied by their surrounding microenvironment^1,11^ or by differentiation factors like retinoic acid^11^. Notably, fMLP receptors can be up-regulated by cytokines and differentiation factors like retinoic acid^53^. For example, it was found that the number of fMLP receptors in the leukemia cell line U937 increased by 8-fold upon activation by lymphokines and in parallel their chemotaxis response was greatly enhanced^54,55^.

It is noteworthy that the magnitude of the response observed after fMLP challenge did not correlate with f-TRPV2 expression levels found in the leukemic cell lines. In fact, the K562 leukemia cell line which has the highest level of f-TRPV2 displayed the weakest response to fMLP compared to the other leukemia cell lines U937 and THP-1. This discrepancy may be due simply to a difference in expression levels and/or type of fMLP receptors in these leukemia cell lines. Indeed, there are two known functional fMLP receptors^53^, namely FPR (formyl peptide receptor) and FPRL1 (FPR like-1), with high and low affinities for fMLP, respectively. THP-1 cells like human monocytes, do express FPR and FPRL1, and migrate in response to fMLP^53^. However, we are not aware of the type of fMLP receptors expressed in U937 and K562 cells. Nonetheless, it was reported that leukemic blasts directly obtained from CML patients expressed fewer fMLP receptors than normal PBMCs; their chemotactic index (mobility) required fMLP concentrations of 10–100-fold higher than those required for normal PBMCs, but their PAF and LTB4 chemotactic pathways were preserved^56,57^. Hence, it will be of interest to investigate if these signaling pathways are also affected by TL and SKF.

Several clinically relevant studies have shown that leukemic cell growth and proliferation were driven by constitutive activation of phosphatidylinositol 3-kinase (PI3K) and MAPK pathways due to upregulation of autocrine and/or paracrine IGF-I/IGF-IR signaling^58–61^. Importantly, PI3K-induced increase in TRPV2 Ca^2+^ activity^22,30,37^ is required for the IGF-I/IGF-IR^37^ and insulin^30,60^ signaling, which were inhibited by SKF and TL respectively. Therefore, we propose that overexpression of TRPV2 shall represent a key event in the transduction of signal from IGF-1/IGF-1R/PI3K system to downstream pathways (e.g. MAPK) in order to sustain survival and proliferation of leukemic blasts, especially during episodic communication of leukemia cells with IGF-1R ligands released from the surrounding microenvironment.

Another important finding of this study is the ability of SKF and TL to target the surface antigen CD38 and substantially reduce its expression in LBCs. Subsequently, multiple functions linked to this plasma membrane protein will be affected. As a receptor, CD38 is involved in the regulation of transient adhesion events that take place between circulating lymphocytes and endothelial cells in which CD31 (also known as PECAM-1) was identified as a ligand for CD38^43^. Moreover, CD38 functions as an ecto-enzyme converting NAD^+^ into several products including the calcium-mobilizing metabolite cyclic ADP-ribose (cADPR)^43,44^, which has been found to promote chemotaxis of primary peripheral blood human neutrophils and monocytes to FPRL1-ligands other than fMLP^62^. Hence, down-regulation of CD38 expression by SKF and TL could affect a key step in LBCs extravasation into lung parenchyma and interstitium.

Considering the pivotal role of CD38/cADPR pathway in the pathogenesis of inflammatory airway disorders (e.g. asthma) through regulation of immune cells chemotaxis and inflammatory reactions in response to environmental stimuli (e.g. infectious agents, allergens)^63^, it could be speculated that, in resident cells of the lung, this signaling pathway may also become activated during episodes of severe leukemia known for its susceptibility to hyperinflammation (e.g. infection). Therefore, CD38/cADPR/FPRL1 and fMLP/TRPV2/FPR(L1) could be either parallel or alternative chemotactic signaling pathways by which immature and functionally incompetent LBCs infiltrate the lung, accumulate in large numbers and contribute to pulmonary dysfunction. Interestingly, TL, which was approved decades ago for the treatment of inflammatory diseases (e.g. bronchial asthma, allergic rhinitis) with remarkable success^31^, could be targeting the CD38/cADPR pathway by down-regulating CD38 expression.

In this study, we used an *in vitro* model that recapitulates somewhat the *in vivo* situation where LBCs are found in the extracellular spaces of lung parenchyma, and accumulate in close proximity to AECs. In this co-culture setting, we set out to prove that LBCs could affect AECs bioelectrical properties. Evidence sustaining this hypothesis has been collected from our electrophysiological studies which demonstrated a discrete and invasive behavior of LBCs towards AECs by disrupting their physical barrier. Indeed, we recorded a drastic and persistent decrease in AEC electrical resistance (i.e. increase in ion conductance) without a statistically significant change in short-circuit current (i.e. transcellular basal active ion transport). The tight-junction paracellular pathway is the major determinant of transepithelial resistance (conductance) in leaky epithelia such as airway epithelium^14^. We previously established that under normal conditions, tight junctions (TJs) restrict paracellular diffusion of small solutes and fluid across the epithelium and constitute an important determinant of how the airways respond to the environment^14^. Thus, alteration of the TJs paracellular pathway by LBCs shall result in increased paracellular permeability due very likely to impairment of TJs structural and functional organization. In fact, in an elegant study using primary human brain microvascular endothelial cells as an *in vitro* model of blood brain barrier (BBB) and a mouse model of CNS leukemia, Feng and co-workers^64^ demonstrated the ability of matrix metalloproteinases MMP-2 and MMP-9, secreted by leukemic cells, to induce a breakdown of the BBB by down-regulating TJs proteins and altering their architecture. In another study, MMP9 was found to evoke mislocalization of TJs proteins leading to increased transepithelial electrical conductance across AECs^15^. Thus, we may suspect that LBCs derived MMPs may contribute to the loss of AECs electrical barrier integrity given the fact that MMP-2/-9 are associated with the invasive behavior of leukemic cells^65^, and can be carried out as messenger RNA transcripts in exosomes^66^ that mediate communication of LBCs with its environment in order to promote their survival and proliferation. Especially, exosomes are able to travel in extracellular spaces and deliver their cargo to distant cells and impact their structure and function^1,66^. Clinically, such event will promote airway remodeling, and this may in turn weakens the defense mechanisms of airway epithelium such as the mucociliary clearance of inhaled particles (e.g. allergens, viruses, and bacteria), increases susceptibility to infection or the risk for acquiring secondary infections, ultimately leading to exacerbated lung inflammation and respiratory failure. This clinical scenario is likely to occur during aggressive (advanced) stages of leukemia with a high blast cell counts (up to 70 to 90%), and LBCs settled in the extravascular spaces of the lungs^4,7^ that can predispose patients to a range of radiologically detected pulmonary complications (e.g. pneumonia, pulmonary hemorrhage, or edema)^4,7^. However, it is important to keep in mind that the patient’s immunocompromised status and chemotherapeutic medications (e.g. ATRA, TKIs) may also contribute to the aforementioned pulmonary abnormalities^4,7^. Therefore, it is important to recognize LBCs infiltration of the lungs as an important event during the course of leukemia progression as they may directly or indirectly contribute to infectious as well as noninfectious pulmonary complications.

Ussing chambers studies revealed that TL and SKF, applied from basolateral and apical sides respectively, were able to stimulate ion transport across AECs regardless of their application order and whether they were applied before or after F/I indicating that these drugs are acting on different ion channel systems and/or signaling molecules that are awaiting discovery.

Most importantly, a long-term treatment with TL prevented LBCs-induced AECs electrical barrier integrity without affecting active ion transport across AECs as judged by their response to cAMP-agonists (i.e. CFTR activators). In fact, TRPV2 overexpression was reported to enhance cell migration by inducing up-regulation of MMP-2, MMP-9, and cathepsin B in a xenograft mouse model of metastatic prostate cancer^20^. Moreover, TL was found to inhibit TRPV2-controlled exocytotic processes^10,60^, and suppress expression, release, and activity of MMP-2 and MMP-9 in a variety of cell types^67–69^. Thus, it is possible that TL prevents damage to airway epithelium by inhibiting these proteases through targeting the expression profile of TRPV2 and the vesicular trafficking pathways associated with this channel.

As a pleiotropic anti-inflammatory drug, TL was also found to target other inflammatory mediators that might be implicated in leukemic infiltration of the lung^65^. For example, TL was found to inhibit the release of IL-8, expression of intercellular adhesion molecule-1 (ICAM-1), and vascular cell adhesion molecule-1 (VCAM-1) in human corneal fibroblasts^67^; interestingly, leukemic cells derived exosomes were found to elicit an increase in the expression of these molecules in human vascular endothelial cells^66^. Hence, other inflammatory components may also contribute to the breakdown of AECs barrier function.

Collectively, these results show that immature LBCs retain the ability to alter airway epithelium barrier function, and suggest that infiltration of LBCs into the lung is clinically more relevant to leukemia than it was taught before. Because LBCs and AECs did not physically interact with each other, there must be secreted factor(s) either from LBCs, AECs, or both that are directly or indirectly compromising the integrity of the airway epithelium. Future studies will certainly shed light on these factors, the TJs proteins affected, the extent of paracellular permeability, and the mechanism(s) by which TL and SKF stimulate active ion transport across AECs.

## Conclusion

Overall, our findings demonstrated that TRPV2 exhibits an oncogenic activity in LBCs that is associated with alteration of its molecular expression profile. Targeting TRPV2 could affect the signaling pathways associated with LBCs growth/proliferation and chemotaxis/infiltration processes, prompting the evaluation of TRPV2 as a promising pharmacodynamic biomarker especially in the setting of aggressive stages of leukemia that might be associated with high risk of [lung] hyperinflammation due to LBCs infiltration. However, further studies using patients’ samples are needed to validate TRPV2 as a reliable molecular biomarker with clinical utility that can guide patient care toward the choice of ideal populations that might benefit from a novel therapeutic approach. TL was effective in all leukemic cell lines tested here, and surprisingly primary blast of some leukemia patients also responded well to TL treatment^70^, warranting its further pre-clinical assessment as stand-alone treatment or combination regimens with other currently used standard chemotherapeutic drugs to pave the way for proof-of-concept clinical studies. Many years of clinical use of TL as an anti-inflammatory agent for airway diseases may benefit leukemia patients that are at high risk for developing (or suffering from) pulmonary complications and underscoring the importance of translation of this therapeutic approach to the clinic.

## Methods

### Reagent and antibodies

SKF96365 (S7809-5MG), Tranilast (T0318-10MG), N-Formyl-Met-Leu-Phe (fMLP, F3506-10MG), Forskolin (F6886-10MG), 3-Isobutyl-1-methylxanthine (IBMX, I5879-100MG), and all the other chemicals are procured from Sigma unless otherwise stated.

### Leukemia cell lines

K562 (French–American–British [myeloid leukemia classification] (FAB) M1 subtype) is a Chronic myelogenous (Myeloid) leukemia (CML) cell line that harbors the Bcr-Abl fusion protein, and U937 (FAB M5 subtype) cells harbor a t(10;11) translocation found in acute monocytic leukemia cells and T cell acute lymphoblastic leukemia^46^. THP-1 (FAB M5 subtype) is an acute monocytic leukemia cell line characterized as a spontaneously immortalized monocyte-like cell line and harboring a t(9;11) translocation^71^.

K562, U937, and THP-1 were maintained in RPMI 1640 containing 10% fetal bovine serum (FBS), penicillin (100 U/ml), and streptomycin (100 µg/ml) at 37 °C in humidified atmosphere containing 5% CO_2_.

### Lung airway epithelial cells

The Calu-3 cell line was obtained from the American Type Culture Collection (ATCC® HTB55™). Calu-3 cells were grown in Dulbecco’s modified Eagle’s medium (DMEM, 31966047, ThermoFisher), supplemented with 10% FBS, penicillin (100 U/ml), streptomycin (100 µg/ml), in a humidified atmosphere containing 5% CO_2_ at 37 ^o^C.

### Differentiation of calu-3 cells

Calu-3 cells were seeded onto 12 mm Snapwell Inserts (CLS3407, Sigma) at density of 1.5 × 10^6^ cells per insert with 2 ml of culture medium added to basolateral side. The following day, residual medium bathing apical side of insert was removed to establish air interface-based culture conditions (ALI). Cells were fed every 48 h from basolateral side and typically after 10–20 days of culture under ALI, cells formed a highly differentiated confluent monolayer as judged by measuring transepithelial electrical resistance (TER; Ω.cm^2^) and spontaneous transepithelial potential difference (p.d.; mV), using a “chopstick” voltmeter (Millicell ERS-2; Millipore).

### Co-culture

Differentiated calu-3 cells were co-cultured with K562, U937, or THP-1 cells added to basolateral compartment at density of 3 × 10^6^ cells per insert in 2 ml of complete DMEM for 48 h prior to measuring bioelectrical properties. Note that an initial assessment of LBCs culture in DMEM for weeks showed a similar or better growth than RPMI.

### RNA isolation and Real-time quantitative PCR (qRT-PCR) analysis of TRPV2

Total RNA was extracted from samples using TRIzol Reagent Ambion RNA (ThermoFisher**)** following manufacturer’s protocol. Concentration and purity of RNA samples were determined by Nanodrop (ND-1000, ThermoFisher). Total RNA (2 μg) was reverse transcribed (RT) with High capacity cDNA reverse transcription kit (ThermoFisher**)** according to manufacturer’s protocol. qRT-PCR of TRPV2 mRNA transcript was performed using TaqMAN gene expression assay kit (Applied Biosystems-4351372). GAPDH (Applied Biosystems-003412) was used as internal control. For qRT-PCR amplification of TRPV2 short form, a specific primer Forward 5′-gctggctgaacctgctttac-3′ and reverse 5′-ctcgagagttcgagggacac-3′ was used to amplify this splice region. Cyber green master mix was used for the amplification reaction. Samples were assayed in triplicates in the same plate using β-Actin as an endogenous control. TRPV2 levels were calculated by the 2-ΔΔCT method, and expressed as relative fold compared to corresponding control.

### Western blot analysis

Whole cell lysates were prepared in Laemmli sample buffer (2×) supplemented with 5% 2-mercaptoethanol and loaded onto 10 or 12% SDS-PAGE gel for protein separation by electrophoresis for 1–3 h at 80–100 V and transfer onto polyvinylidene difluoride membranes (Immobilon, Millipore) for 1 h at 120 V. The membranes were blocked in 5% (wt/vol) nonfat milk 0.1% Tween TBS buffer for 1 h at room temperature, and probed overnight at 4 ^o^C with one of the following primary antibodies: anti-TRPV2 (OSR00190W, ThermoFisher), anti-PARP (9532), anti-caspase-3 (3-9665), anti-cleaved caspase-3 (3-9664), anti-Bcl-2 (15071), anti-ERK1/2 (4695), anti-phospho-ERK1/2 (N^o^ 3179), anti-p38 (N^o^ 8690), anti-phospho-p38 (4511), anti-β-tubulin (2146), and anti-GAPDH (N^o^ 2118). The membranes were incubated with horseradish peroxidase-linked secondary antibodies and visualized for immunoreactivity using enhanced chemiluminescence kit and Chemidoc system (Amersham, Bio-Rad). Antibodies are from Cell signaling except otherwise specified.

### TRPV2 silencing

Leukemia cell lines were transfected with fluorescein-coupled small interfering RNA (siRNA) against TRPV2 (Hs_TRPV2_5 FlexiTube siRNA SI02781359, Qiagen) by electroporation using Neon Transfection System (MPK5000, ThermoFisher) and Neon kit (MPK10096, ThermoFisher). Non-targeting siRNA (negative control siRNA, 1027310, Qiagen) was used as control. After 24 h, transfection efficiency was determined by qRT-PCR, Western blot, and fluorescence microscopy (Olympus inverted microscope IX51, Objective x10/0.25, Olympus).

### Cell proliferation using MTT assay

LBCs were seeded onto 96-well plates at a density of 1 × 10^4^ cells per well and incubated with TL or SKF at the indicated concentrations at 37 °C in CO_2_-incubator for the designated time periods. Cell mortality was determined using the Vybrant MTT cell proliferation assay kit (M6494, ThermoFisher). All drug concentrations were tested at least in triplicate wells and the assay was performed at least in three separate experiments. The optical density (OD) of each well was measured using an ELISA plate reader at 570 nm. After blank subtraction, the percentage of cell viability was calculated according to the following equation:

$${\rm{Cell}}\,{\rm{viability}}\,{\rm{index}}( \% )=[{{\rm{OD}}}_{570}({\rm{sample}})/{{\rm{OD}}}_{570}({\rm{control}})]\times 100$$

### Assessment of mitochondrial membrane potential

Mitochondrial membrane depolarization occurring in apoptosis was evaluated using JC-1 dye (T3168, ThermoFisher). LBCs were seeded onto 24- or 48-well plates at a density of 2 × 10^5^ cells per well, and incubated for 48 h with TL or SKF at the indicated doses at 37 °C in CO_2_-incubator. Then, cells were stained with 2 μM JC-1 for 30 min at 37 °C, and analyzed by Flow Cytometry (BD LSRFortessa Cell Analyzer, BD Biosciences) using 488 nm excitation with 530/30 or 585/42 nm bypass emission filters. The fraction of cells manifesting mitochondrial membrane depolarization is determined from the ratio of JC-1 green and red fluorescence.

### Detection of apoptosis

Annexin V coupled either to APC (Annexin V-APC, *Annexin A5-Allophycocyanin*, 550474, BD Biosciences) or to BV421 (Annexin V- BV421, *Annexin A5- BD Horizon™ BV421*, BD Biosciences) and Propidium Iodide (PI) Staining Solution (556463, BD Biosciences) were used to detect early and late apoptosis respectively. Leukemic cells (2 × 10^5^ cells/well) were treated with SKF or TL as described before for 48 h and co-stained with Annexin V-APC/BV421 (5 µL/sample) and PI (0.5 µL/sample) in Annexin binding buffer (556454, BD Biosciences, 100 µL/sample) for 20 min at room temperature. Then, cells were analyzed by flow cytometry to quantify Live (Annexin V-APC/BV421 −ive, PI −ive), Early Apoptotic (Annexin V-APC/BV421 +ive, PI −ive), Late Apoptotic (Annexin V-APC/BV421 +ive, PI +ive) and Necrotic (Annexin V-APC/BV421 −ive, PI +ive) cell fractions.

### Analysis of cell cycle distribution

Cells in different phases of the cell cycle were distinguished based on the fluorescence intensity of the stoichiometric DNA binding dye, Hoechest 33342. Leukemic cells (0.2 × 10^6^ cells/well) were treated with indicated doses of TL or SKF for 48 h at 37 °C. The cells will be incubated with Hoechst 33342 (BD Biosciences, 0.1 µg/0.5 mL) for 20 min at 37 °C. After incubation, the cells were washed extensively with Phosphate Buffer Solution (PBS) and then analyzed for DNA content by Flow Cytometry.

### Intracellular Ca^2+^ measurements

Changes in intracellular Ca^2+^ ([Ca^2+^]_i_) were measured using the cell permeable Ca^2+^ probe Fluo-4 AM (F14201, ThermoFisher). LBCs were seeded onto 24- or 48-well plates in cell culture media at a density of 2 × 10^5^ cells per well, and incubated for 48 h with TL or SKF at the indicated doses at 37 °C in CO_2_-incubator. Next, cells were loaded with 10 µM Fluo-4 AM in presence of 0.02% pluronic acid F-127 (P2443, Sigma) at 37 °C for 45 min, and incubated for an additional 20 min to allow complete de-esterification of intracellular AM esters prior to analysis by flow cytometry using 488 nm excitation with 530/30 nm bypass emission filter. Each value of the data is presented as fluorescence index of the probe (Ratio of treated to control fluorescence normalized intensities).

### Analysis of CD38 cell surface expression

After a treatment with TL or SKF for 48 h (K562 and U937 cells) or 96 h (THP-1 cells) as described before, LBCs (1 × 10^6^) were incubated with CD38-BB515 antibody (N^o^ 564498, BD Biosciences) diluted in staining buffer (FBS) (554656, BD Biosciences) for 30 min at 37 °C, and analyzed by flow cytometry using 488 nm excitation with 530/30 nm bypass emission filter to measure CD38 expression level in live cells.

### Ussing chambers experiments

Snapwell inserts were mounted between half-chambers (Physiologic Instruments) separating mucosal and serosal bathing solutions of identical ionic composition. Both sides of the tissue are bathed in 5 ml bicarbonate-buffered Krebs-Henseleit solution, which contained (in mM) 126 NaCl, 0.38 KH_2_PO4, 2.13 K_2_HPO_4_, 1.2 MgCl_2_, 1.2 CaCl_2_, 25 NaHCO_3_, and 10 glucose; osmolarity of this solution was ~320 mosm, and pH was 7.4 when bubbled with 95% O_2_–5% CO_2_ at 37 °C. Calu-3 monolayers were continuously short-circuited (membrane potential clamped to zero) after fluid resistance compensation using a Physiologic Instruments voltage clamp VCC-MC6. The short-circuit current (I_sc_) (amount of electrical current needed to maintain voltage clamp to zero) was recorded on a computer using Physiologic Instruments interface and “Acquire & Analyze” software (Version 2.3). I_sc_ represents the sum of all active ion transports across the tissue^12^. Transepithelial resistance (TER) was measured at 10-s intervals from the deflections in current caused by constant voltage pulses (620 ms duration, 5 mV bipolar pulse). A waiting time period of 15–20 min is usually required for the baseline I_sc_ and TER to stabilize before applying drugs as aliquots of 100- or 1000-fold concentrated stock solutions.

### Data Analysis

Experiments were performed at least in triplicate per condition and repeated at least three times. Data were expressed as the mean ± SD (standard deviation) or SEM (standard error of the mean). Statistical analysis was performed by 2-sided Student’s t-test or one-way ANOVA followed by post hoc comparisons using the 2-sided Student’s t-test with the Bonferroni correction. Differences between experimental conditions resulting in p < 0.05 were considered statistically significant.

## Supplementary information

Supplementary figures and Table

## Data Availability

All data generated or analyzed during this study are included in this published article (and its Supplementary Information files).

## References

[1] Bakker E, Qattan M, Mutti L, Demonacos C, Krstic-Demonacos M (2016). The role of micro-environment and immunity in drug response in leukemia. Biochim. Biophys. Acta.

[2] Konopleva MY, Jordan CT (2011). Leukemia Stem Cells and Microenvironment: Biology and therapeutic targeting. J. Clin. Oncol..

[3] Chandran, R., Hakki, M. & Spurgeon, S. Infections in leukemia, Sepsis Luciano Azevedo, IntechOpen, 10.5772/50193 (2012).

[4] Koh TT, Colby TV, Muller NL (2005). Myeloid leukemias and lung involvement. Semin. Respir. Crit. Care Med..

[5] Lamour C, Bergeron A (2011). Non-infectious pulmonary complications of myelodysplastic syndromes and chronic myeloproliferative disorders. Rev. Mal. Respir..

[6] Rosenow EC, Wilson WR, Cockerill FR (1985). Pulmonary disease in the immunocompromised host. 1. Mayo Clin. Proc..

[7] Hildebrand FL, Rosenow EC, Habennann TM, Tazelaar HD (1990). Pulmonary complications of leukemia. Chest.

[8] Schroeder MA, DiPersio JF (2012). Mobilization of hematopoietic stem and leukemia cells. J. Leukoc. Biol..

[9] Song JH (2009). Enhanced invasiveness of drug-resistant acute myeloid leukemia cells through increased expression of matrix metalloproteinase-2. Int. J. Cancer.

[10] Baba A (2016). Anti-allergic drugs tranilast and ketotifen dose-dependently exert mast cell stabilizing properties. Cell. Physiol. Biochem..

[11] Luesink M, Jansen JH (2010). Advances in understanding the pulmonary infiltration in acute promyelocytic leukaemia. British J. Haematol..

[12] Azizi F, Arredouani A, Mohammad RM (2015). Airway surface liquid volume expansion induces rapid changes in amiloride-sensitive Na^+^ transport across upper airway epithelium-Implications concerning the resolution of pulmonary edema. Physiol. Rep..

[13] Hiemstra PS, McCray PB, Bals R (2015). The innate immune function of airway epithelial cells in inflammatory lung disease. Eur. Respir. J..

[14] Azizi F, Matsumoto PS, Wu DX, Widdicombe JH (1997). Effects of hydrostatic pressure on permeability of airway epithelium. Exp. Lung Res..

[15] Vermeer PD (2009). MMP9 modulates tight junction integrity and cell viability in human airway epithelia. Am. J. Physiol. Lung Cell Mol. Physiol..

[16] Santoni G, Farfariello V, Amantini C (2011). TRPV channels in tumor growth and progression. Adv. Exp. Med. Biol.

[17] Morelli MB (2013). Expression and function of the transient receptor potential ion channel family in the hematologic malignancies. Curr. Mol. Pharmacol..

[18] Santoni, G., Farfariello, G. & Amantini, C. Chapter 49: TRPV channels in tumor growth and progression. (ed. Islam, M. S.). Transient receptor potential channels, Adv. Exp. Med. Biol. 704 (2011). 10.1007/978-94-007-0265-3_49 21290335

[19] Elbaz, M. *et al*. TRPV2 is a novel biomarker and therapeutic target in triple negative breast cancer. *Oncotarget* **27** (2016). 10.18632/oncotarget.9663 PMC6173360 30323891

[20] Monet M (2010). Role of cationic channel TRPV2 in promoting prostate cancer migration and progression to androgen resistance. Cancer Res..

[21] Liberati S (2014). Loss of TRPV2 homeostatic control of cell proliferation drives tumor progression. Cells.

[22] Peralvarez-Marin A, Donate-Macian P, Gaudet R (2013). What do we know about the transient receptor potential vanilloid 2 (TRPV2) ion channel?. FEBS J..

[23] Santoni G (2013). The role of transient receptor potential vanilloid type-2 ion channels in innate and adaptive immune responses. Front. Immunol..

[24] Nagasawa M, Nakagawa Y, Tanaka S, Kojima I (2007). Chemotactic Peptide fMetLeuPhe Induces Translocation of the TRPV2 Channel in Macrophages. J. Cell Physiol..

[25] Link. (2010). TRPV2 has a pivotal role in macrophage particle binding and phagocytosis. Nat. Immunol..

[26] Issa CM (2014). TRPV2 in the Development of Experimental Colitis. Scand. J. Immunol..

[27] Nie L, Oishi Y, Doi I, Shibata H, Kojima I (1997). Inhibition of proliferation of MCF-7 breast cancer cells by a blocker of Ca(2+)-permeable channel. Cell Calcium.

[28] Mihara H (2010). Involvement of TRPV2 Activation in Intestinal Movement through Nitric Oxide Production in Mice. J. Neurosci..

[29] Aoyagi K, Ohara-Imaizumi M, Nishiwaki C, Nakamichi Y, Nagamatsu S (2010). Insulin/phosphoinositide 3-kinase pathway accelerates the glucose-induced first-phase insulin secretion through TrpV2 recruitment in pancreatic beta-cells. Biochem. J..

[30] Hisanaga E (2009). Regulation of calcium-permeable TRPV2 channel by insulin in pancreatic β-cells. Diabetes.

[31] Darakhshan S, Pour AB (2015). Tranilast: a review of its therapeutic applications. Pharmacol. Res..

[32] Pottosin I (2015). Mechanosensitive Ca(2)(+)-permeable channels in human leukemic cells: pharmacological and molecular evidence for TRPV2. Biochim. Biophys. Acta.

[33] Leung Y-M, Kwan C-Y, Loh T-T (1996). Dual effects of SK&F 96365 in human leukemic HL-60 cells: Inhibition of calcium entry and activation of a novel cation influx pathway. Biochem Pharmacol.

[34] Yang S, Zhang JJ, Huang XY (2009). Orai1 and STIM1 are critical for breast tumor cell migration and metastasis. Cancer Cell.

[35] Stokes AJ, Shimoda LMN, Koblan-Huberson M, Adra CN, Turner HA (2004). TRPV2–PKA Signaling Module for Transduction of Physical Stimuli in Mast Cells. J. Exp. Med..

[36] Cohen MR (2015). Nerve growth factor regulates transient receptor potential vanilloid 2 via extracellular signal-regulated kinase signaling to enhance neurite outgrowth in developing neurons. Mol. Cell Biol..

[37] Reichhart N, Keckeis S, Fried F, Fels G, Strauss O (2015). Regulation of surface expression of TRPV2 channels in the retinal pigment epithelium. Graefes Arch. Clin. Exp. Ophthalmol..

[38] Zhang D (2012). Mast-Cell Degranulation Induced by Physical Stimuli Involves the Activation of Transient-Receptor-Potential Channel TRPV2. Physiol. Res..

[39] Indran IR, Tufo G, Pervaiz S, Brenner C (2011). Recent advances in apoptosis, mitochondria and drug resistance in cancer cells. Biochim. Biophys. Acta.

[40] Kuo LJ, Yang L-X (2008). γ-H2AX – A novel biomarker for DNA double-strand breaks. In vivo.

[41] Porras A, Guerrero C (2011). Role of p38α in apoptosis: implication in cancer development and therapy. Atlas Genet. Cytogenet. Oncol. Haematol..

[42] Orrenius S, Zhivotovsky B, Nicotera P (2003). Calcium: Regulation of cell death: the calcium–apoptosis link. Nat. Rev. Mol. Cell Biol..

[43] Van de Donk NWCJ (2016). Monoclonal antibodies targeting CD38 in hematological malignancies and beyond. Immunol. Rev..

[44] Wei W, Graeff R, Yue J (2014). Roles and mechanisms of the CD38/cyclic adenosine diphosphate ribose/Ca^2+^ signaling pathway. World J. Biol. Chem..

[45] Voermans C, Van Heese WPM, De Jong I, Gerritsen WR, Van der Schoot CE (2002). Migratory behavior of leukemic cells from acute myeloid leukemia patients. Leukemia.

[46] Jensen HA, Yourish HB, Bunaciu RP, Varner JD, Yen A (2015). Induced myelomonocytic differentiation in leukemia cells is accompanied by noncanonical transcription factor expression. FEBS Open Bio..

[47] Spinsanti G (2008). Quantitative Real-Time PCR detection of TRPV1–4 gene expression in human leukocytes from healthy and hyposensitive subjects. Mol. Pain.

[48] Zhou K, Zhang S-S, Yan Y, Zhao S (2014). Overexpression of transient receptor potential vanilloid 2 is associated with poor prognosis in patients with esophageal squamous cell carcinoma. Med. Oncol..

[49] Nabissi MM (2010). TRPV2 channel negatively controls glioma cell proliferation and resistance to Fas-induced apoptosis in ERK-dependent manner. Carcinogenesis.

[50] Wang C, Hu HZ, Colton CK, Wood JD, Zhu MX (2004). An alternative splicing product of the murine TRPV1 gene dominant negatively modulates the activity of TRPV1 channels. J. Biol. Chem..

[51] Zhang W (2003). A novel TRPM2 isoform inhibits calcium influx and susceptibility to cell death. J. Biol. Chem..

[52] Jones GE (2000). Cellular signaling in macrophage migration and chemotaxis. J. Leukoc. Biol..

[53] Resnati M (2002). The fibrinolytic receptor for urokinase activates the G protein-coupled chemotactic receptor FPRL1/LXA4R. Proc. Natl. Acad. Sci. USA.

[54] Pike MC, Fischer DG, Koren HS, Snyderman R (1980). Development of specific receptors for N-formylated chemotactic peptides in a human monocyte cell line stimulated with lymphokines. J. ExP. MeD..

[55] Hams PE (1985). Distinct Differentiation-inducing Activities of ϒ-lnterferon and Cytokine Factors Acting on the Human Promyelocytic Leukemia Cell Line HL-601. Cancer Res.

[56] Radhika V (1996). Granulocytes From Chronic Myeloid Leukemia (CML) Patients Show Differential Response to Different Chemoattractants. Am. J. Hematol..

[57] Naik NR, Advani SH, Bhisey AN (1989). PMN cells from chronic myeloid leukemia (CML) patients show defective chemotaxis in remission. Leuk. Res..

[58] Yaktapour N (2013). Insulin-like growth factor-1 receptor (IGF1R) as a novel target in chronic lymphocytic leukemia. Blood.

[59] Cohen DH, LeRoith D (2012). Obesity, type 2 diabetes, and cancer: the insulin and IGF connection. Endocr Relat Cancer.

[60] Aoyagi K, Ohara-Imaizumi M, Nishiwaki C, Nakamichi Y, Nagamatsu S (2010). Insulin/phosphoinositide 3-kinase pathway accelerates the glucose-induced first-phase insulin secretion through TrpV2 recruitment in pancreatic β-cells. Biochem. J..

[61] Lichtman M (2010). Obesity and the Risk for a Hematological Malignancy: Leukemia, Lymphoma, or Myeloma. The Oncologist.

[62] Partida-Sánchez S (2004). Chemotaxis and calcium responses of phagocytes to formyl peptide receptor ligands is differentially regulated by cyclic ADP ribose. J. Immunol..

[63] Guedes AGP (2015). CD38 and Airway hyper-responsiveness: Studies on human airway smooth muscle cells and mouse models. Can. J. Physiol. Pharmacol..

[64] Feng S (2011). Matrix metalloproteinase-2 and -9 secreted by leukemic cells increase the permeability of blood-brain barrier by disrupting tight junction proteins. PLoS ONE.

[65] Yu XF, Han ZC (2006). Matrix metalloproteinases in bone marrow: roles of gelatinases in physiological hematopoiesis and hematopoietic malignancies. Histol. Histopathol..

[66] Pando A, Reagan JL, Quesenberry P, Fast LD (2018). Extracellular vesicles in leukemia. Leuk. Res..

[67] Liu Y (2016). Inhibitory effects of tranilast on cytokine, chemokine, adhesion molecule, and matrix metalloproteinase expression in human corneal fibroblasts exposed to Poly(I:C). Curr Eye Res.

[68] Shimizu T (2006). Effect of tranilast on matrix metalloproteinase production from neutrophils *in-vitro*. J. Pharmacy Pharmacol..

[69] Shimizu T (2005). Suppression of matrix metalloproteinase production in nasal fibroblasts by tranilast, an antiallergic agent, *in Vitro*. Mediators Inflamm..

[70] Suwa S (2015). The tryptophan derivative, tranilast, and conditioned medium with indoleamine 2,3-dioxygenase-expressing cells inhibit the proliferation of lymphoid malignancies. Int. J. Oncol..

[71] Tsuchiya S (1980). Establishment and characterization of a human acute monocytic leukemia cell line (THP-1). Int. J. Cancer.

