# Supplementary material for: Evaluation of cationic channel TRPV2 as a novel biomarker and therapeutic target in Leukemia-Implications concerning the resolution of pulmonary inflammation
Source: Sci Rep. 2019 Feb 7;9:1554. doi: 10.1038/s41598-018-37469-8 (PMC6367460; doi:10.1038/s41598-018-37469-8)
Supplement: Supplementary file 1 — Supplementary figures and Table [file 41598_2018_37469_MOESM1_ESM.pdf]

Evaluation of cationic channel TRPV2 as a novel biomarker and therapeutic target in Leukemia-Implications concerning the resolution of pulmonary inflammation

Kodappully S. Siveen[1#], Kirti S. Prabhu[1#], Aeijaz S. Parray[1#], Maysaloun Merhi[2], Abdelilah Arredouani[3], Mohamed Chikri[3], Shahab Uddin[1], Said Dermime[2], Ramzi M. Mohammad[4], Martin Steinhoff [1], Ibrahim A.M. Janahi[5] & Fouad Azizi[1*]

Figure 3S1_gel 1:

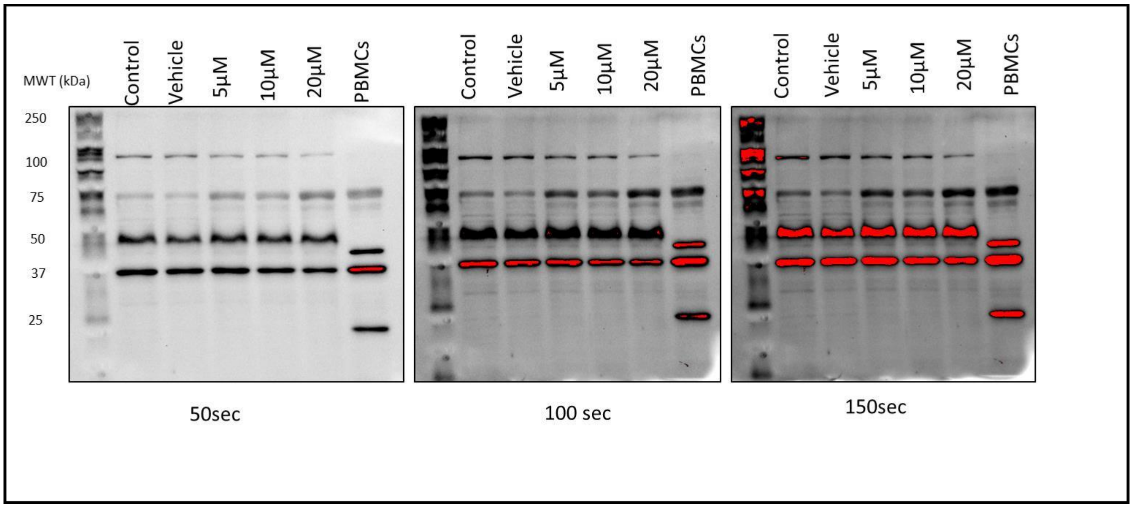

Figure 3S1_gel 2:

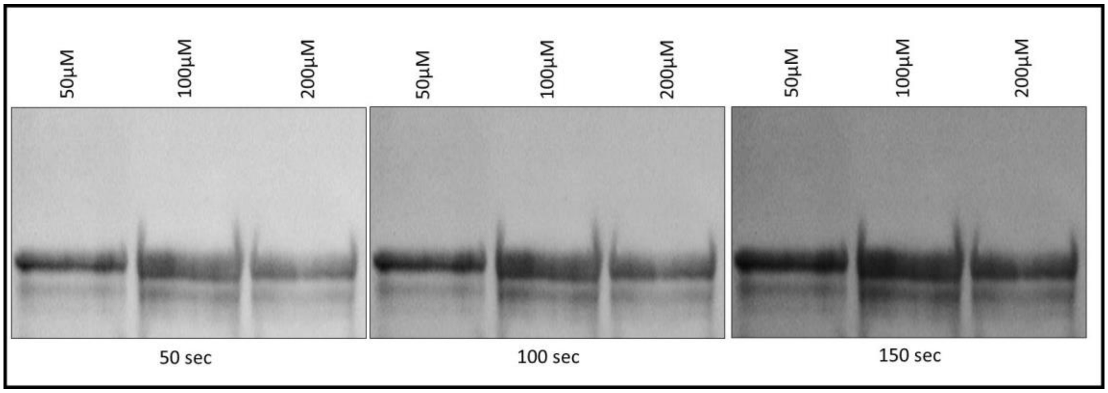

Gels 1 and 2 of SKF samples were run in the same cassette using the same electrophoretic parameters, and processed in the same way afterwards. Equal amounts of protein for SKF samples 50, 100, and 200 µM required higher volumes of cell lysates to be loaded in gel 2 with larger wells

Figure 3S1_gel 3:

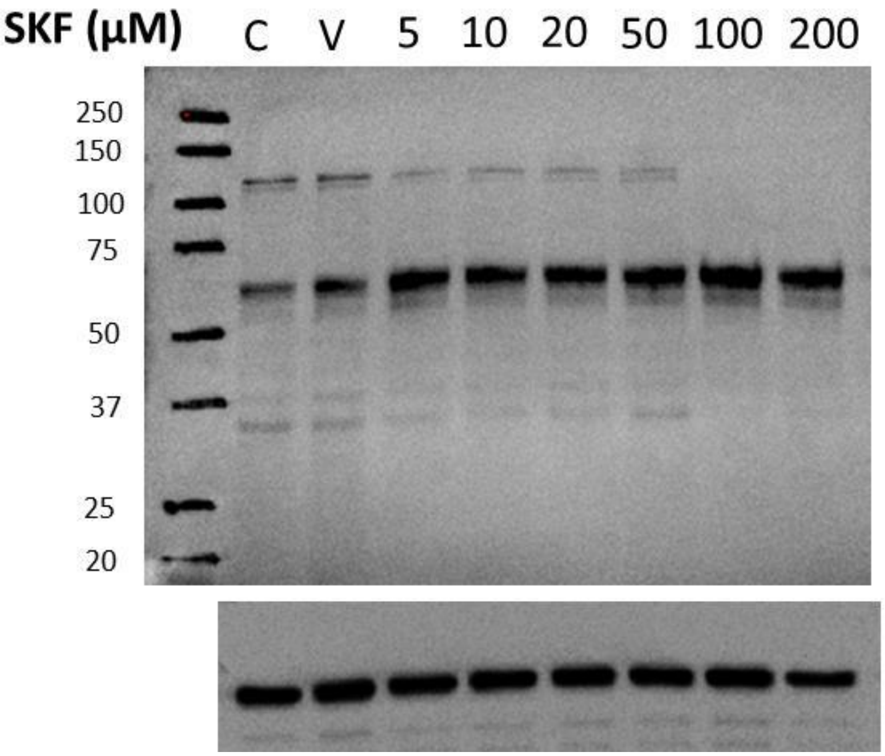

Figure 3S2_gel 1A:

The membrane was cut into two strips which were processed in parallel.

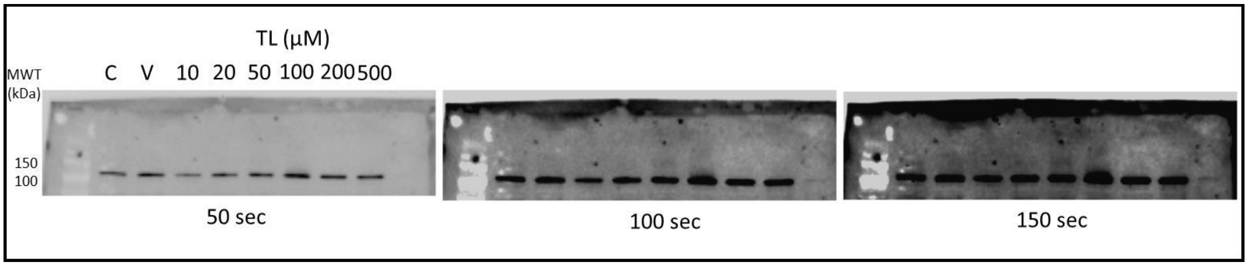

Figure 3S2_gel 1B:

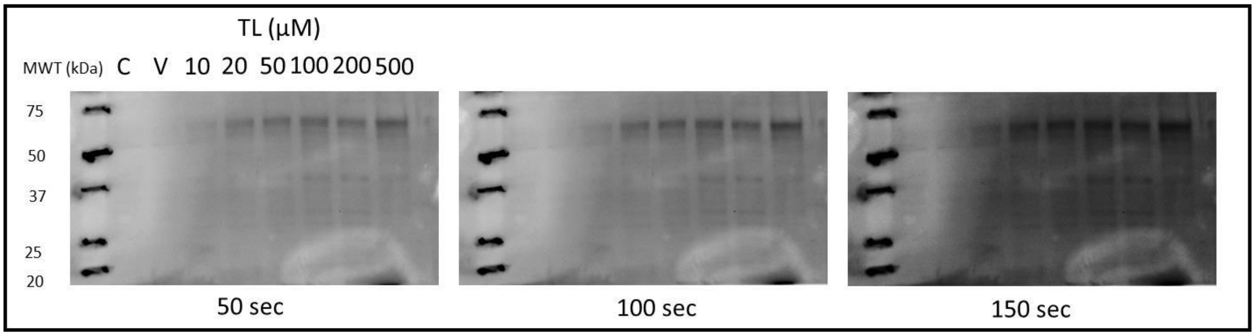

Figure 3S2_gel2:

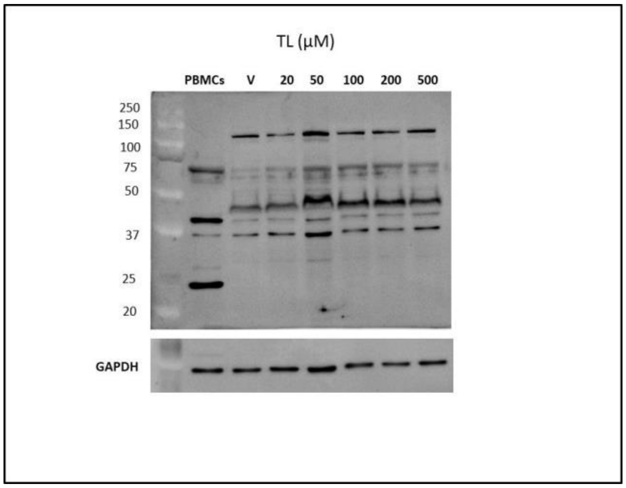

Figure 3S3_gel 1:

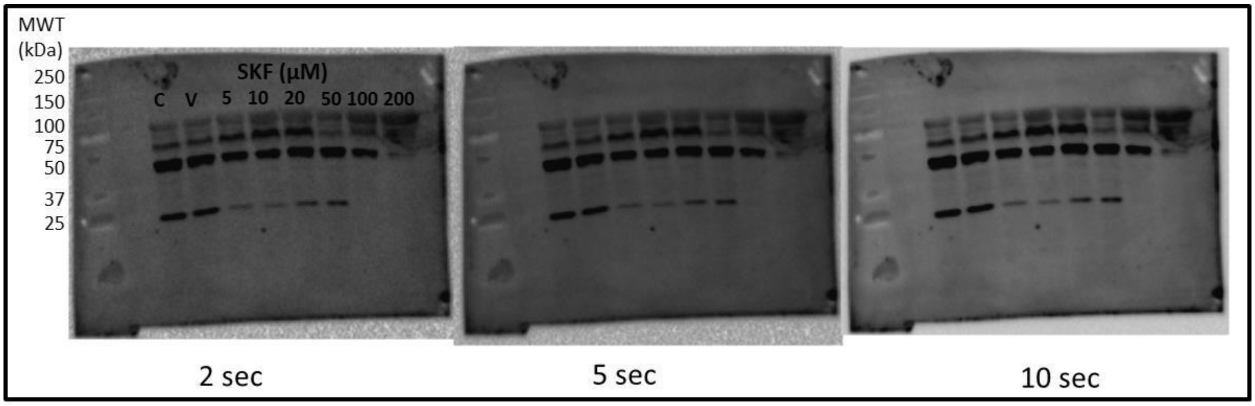

Figure 3S3_gel 2:

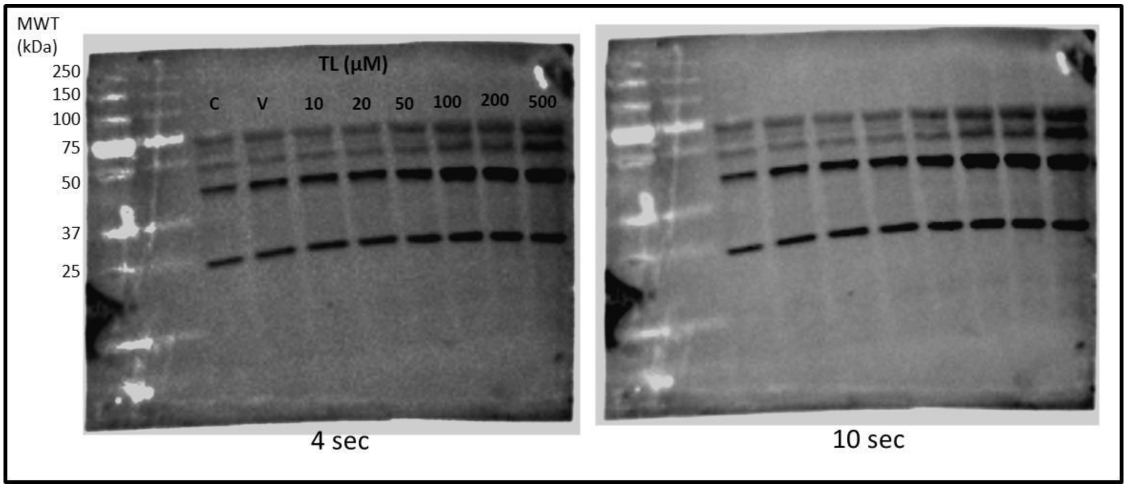

Figure 5S1_gel 1:

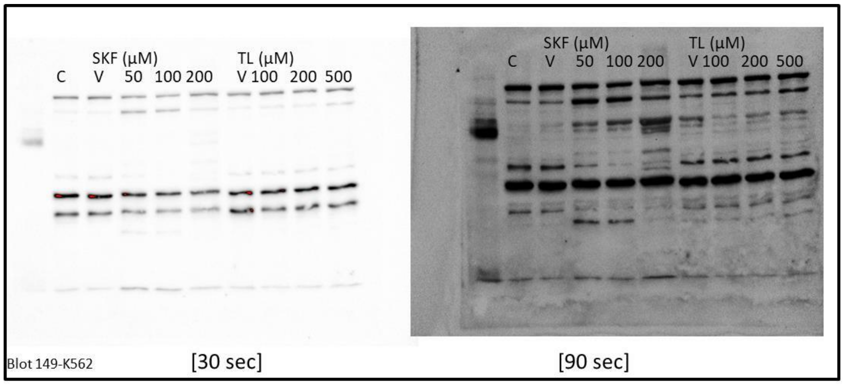

Figure 5S1_gel 2:

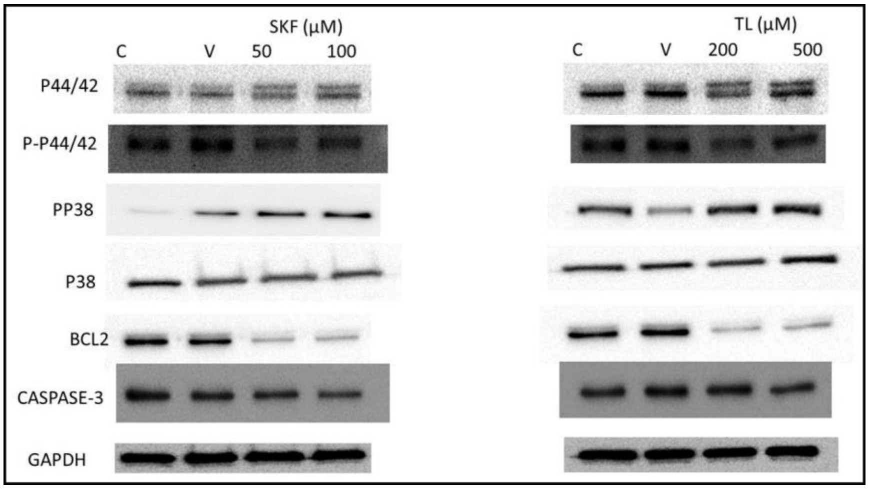

Figure 5S2_gels 1 & 2:

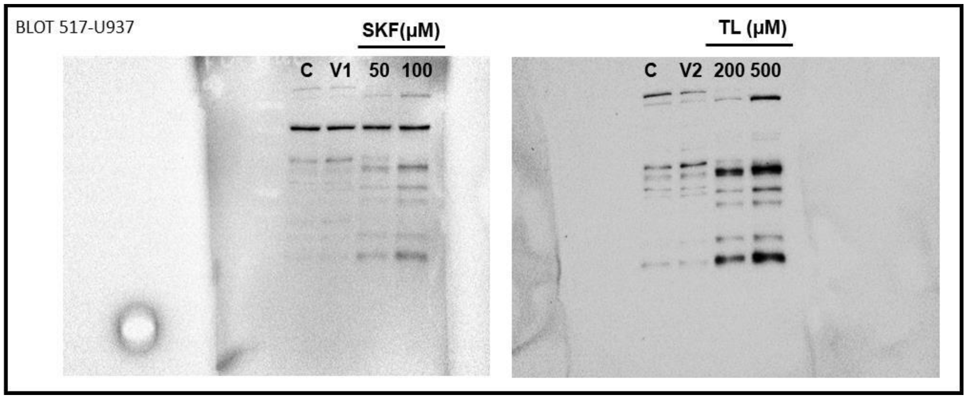

Figure 5S2_gel 3:

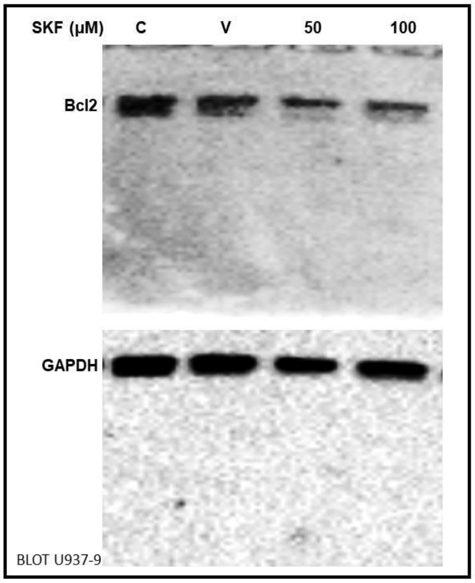

Figure 5S2_gel 4:

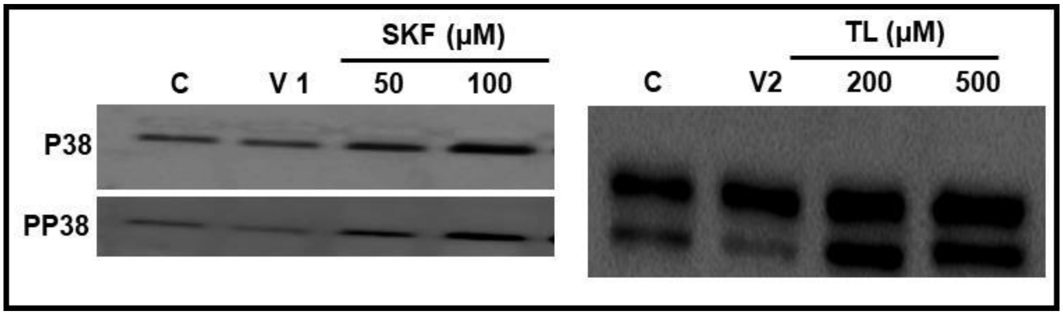

Table 1S. Statistical analysis of drug-induced I_{sc} stimulation in Calu-3.

	**F/I**		**SKF**		**TL**	
Calu-3 (control)	176.4 ± 8.4	(N=9)	220.2 ± 31.7	(N=14)	261.2 ± 77.2	(N=6)
Calu-3 + K562	**115.2 ± 5.0***	(N=3)	222.1 ± 38.1	(N=7)	182.9 ± 34.1	(N=4)
Calu-3 + U937	**141.9 ± 4.3***	(N=6)	267.1 ± 60.9	(N=5)	137.6 ± 15.1	(N=3)
Calu-3 + THP-1	**139.4 ± 10.9***	(N=6)	122.2 ± 7.5	(N=5)	111.6 ± 6.2	(N=3)

Values (mean ± SEM) represent percentage increases from baseline I_{sc} recorded in response to drugs applied at the doses mentioned elsewhere in Fig. 7C. F/I: Forskolin/IBMX (($F(3,20) = 7.7$, $p < 0.001$), *****P < 0.008, significantly different from control).
